# The structural and mechanistic bases for the viral resistance to allosteric HIV-1 integrase inhibitor pirmitegravir

**DOI:** 10.1128/mbio.00465-24

**Published:** 2024-10-15

**Authors:** Tung Dinh, Zahira Tber, Juan S. Rey, Seema Mengshetti, Arun S. Annamalai, Reed Haney, Lorenzo Briganti, Franck Amblard, James R. Fuchs, Peter Cherepanov, Kyungjin Kim, Raymond F. Schinazi, Juan R. Perilla, Baek Kim, Mamuka Kvaratskhelia

**Affiliations:** 1 Division of Infectious Diseases, University of Colorado Anschutz Medical Campus, Aurora, Colorado, USA; 2 Center for ViroScience and Cure, Laboratory of Biochemical Pharmacology, Department of Pediatrics, Emory University School of Medicine, and Children’s Healthcare of Atlanta, Atlanta, Georgia, USA; 3 Department of Chemistry and Biochemistry, University of Delaware, Newark, Delaware, USA; 4 College of Pharmacy, The Ohio State University, Columbus, Ohio, USA; 5 Chromatin Structure & Mobile DNA Laboratory, The Francis Crick Institute, London, United Kingdom; 6 ST Pharm Co., Ltd., Seoul, South Korea; The University of North Carolina at Chapel Hill, Chapel Hill, North Carolina, USA; Academic Medical Center of the University of Amsterdam, Amsterdam, Netherlands

**Keywords:** HIV-1 integrase, antiretroviral drug, ALLINI, pirmitegravir

## Abstract

Allosteric HIV-1 integrase (IN) inhibitors (ALLINIs) are investigational antiretroviral agents that potently impair virion maturation by inducing hyper-multimerization of IN and inhibiting its interaction with viral genomic RNA. The pyrrolopyridine-based ALLINI pirmitegravir (PIR) has recently advanced into phase 2a clinical trials. Previous cell culture-based viral breakthrough assays identified the HIV-1_(Y99H/A128T IN)_ variant that confers substantial resistance to this inhibitor. Here, we have elucidated the unexpected mechanism of viral resistance to PIR. Although both Tyr99 and Ala128 are positioned within the inhibitor binding V-shaped cavity at the IN catalytic core domain (CCD) dimer interface, the Y99H/A128T IN mutations did not substantially affect the direct binding of PIR to the CCD dimer or functional oligomerization of full-length IN. Instead, the drug-resistant mutations introduced a steric hindrance at the inhibitor-mediated interface between CCD and C-terminal domain (CTD) and compromised CTD binding to the CCD_Y99H/A128T_ + PIR complex. Consequently, full-length IN_Y99H/A128T_ was substantially less susceptible to the PIR-induced hyper-multimerization than the WT protein, and HIV-1_(Y99H/A128T IN)_ conferred >150-fold resistance to the inhibitor compared with the WT virus. By rationally modifying PIR, we have developed its analog EKC110, which readily induced hyper-multimerization of IN_Y99H/A128T_ *in vitro* and was ~14-fold more potent against HIV-1_(Y99H/A128T IN)_ than the parent inhibitor. These findings suggest a path for developing improved PIR chemotypes with a higher barrier to resistance for their potential clinical use.

**IMPORTANCE:**

Antiretroviral therapies save the lives of millions of people living with HIV (PLWH). However, the evolution of multi-drug-resistant viral phenotypes is a major clinical problem, and there are limited or no treatment options for heavily treatment-experienced PLWH. Allosteric HIV-1 integrase inhibitors (ALLINIs) are a novel class of antiretroviral compounds that work by a unique mechanism of binding to the non-catalytic site on the viral protein and inducing aberrant integrase multimerization. Accordingly, ALLINIs potently inhibit both wild-type HIV-1 and all drug-resistant viral phenotypes that have so far emerged against currently used therapies. Pirmitegravir, a highly potent and safe investigational ALLINI, is currently advancing through clinical trials. Here, we have elucidated the structural and mechanistic bases behind the emergence of HIV-1 integrase mutations in infected cells that confer resistance to pirmitegravir. In turn, our findings allowed us to rationally develop an improved ALLINI with substantially enhanced potency against the pirmitegravir-resistant virus.

## INTRODUCTION

HIV-1 integrase (IN) is essential for two distinct steps in the virus lifecycle: (i) its enzymatic activities are needed for integration of the double-stranded viral complementary DNA into host cell chromosome, and (ii) during virion morphogenesis, IN binds to the viral RNA genome (vRNA) to ensure proper localization of ribonucleoprotein complexes within the mature capsid. IN is comprised of three domains: the N-terminal domain (NTD), the catalytic core domain (CCD), and the C-terminal domain (CTD, Fig. S1A). Each of these domains is crucial for both catalytic and non-catalytic functions of the viral protein.

The catalytic activity of IN has been exploited as a therapeutic target, and IN strand transfer inhibitors (INSTIs) have been successfully used to treat people living with HIV (PLWH). More recently, allosteric HIV-1 integrase inhibitors (ALLINIs), which target a non-catalytic site on IN, have been developed (1–10). The principal mode of action of ALLINIs is to induce aberrant or hyper-multimerization of the retroviral protein, which is detrimental to both catalytic and non-catalytic functions of IN during early and late steps of HIV-1 replication (3, 11–15). However, in cell culture, ALLINIs much more potently inhibit proper virion maturation than HIV-1 integration (3, 7, 8, 16–18). The cellular cofactor LEDGF/p75, which mediates the integration of HIV-1 into active transcription units (19–21), binds IN at the same non-catalytic dimer interface that is targeted by ALLINIs (22, 23). Accordingly, the competitive interplay between nuclear LEDGF/p75 and ALLINIs during HIV-1 integration substantially reduces the potency of these inhibitors in target cells (24, 25). Overexpression of LEDGF/p75 further decreases ALLINI EC_50_ values, whereas the LEDGF/p75 depletion substantially enhances the potency of these inhibitors during early steps of infection (24). By contrast, during virion morphogenesis, ALLINIs readily induce hyper-multimerization of IN and impair its binding to viral RNA (12). Consequently, the virions produced in the presence of ALLINIs have ribonucleoprotein complexes mislocalized outside of the protective capsid and are non-infectious (3, 7, 12, 16–18, 26–29).

ALLINIs typically contain core aromatic scaffolds, which are flanked by the key pharmacophore carboxylic acid, the *tert*-butoxyl moiety, and halogenated bulky aromatic rings. These inhibitors are anchored to the V-shaped cavity (Fig. S1B) at the IN CCD dimer through an extensive network of hydrogen bonds and hydrophobic interactions (1–3, 7). Biochemical assays have revealed that in addition to the CCD, the NTD and the CTD are crucial for ALLINI-induced aberrant protein multimerization (13, 30). Although how the NTD contributes to the ALLINI-induced protein aggregation remains to be elucidated, recent structural studies have demonstrated that the CTD directly engages with the CCD-ALLINI complex (6, 31–33). Specifically, ALLINIs induce head-to-tail interactions between CCD-CCD of one dimer and the CTD of another dimer (31) (also see Fig. S1C), which leads to the uncontrolled hyper-multimerization of IN, thereby resulting in non-functional protein polymers. The invariant CTD residues engage with both the CCD and the inhibitor to stabilize the CCD-ALLINI-CTD interface. Because ALLINIs are sandwiched between the CCD and the CTD, these inhibitors exhibit a substantially lower *K_off_* rate and a higher affinity (*K_D_*) for the CCD-ALLINI-CTD vs the CCD-ALLINI complex (32).

**Fig 1:**
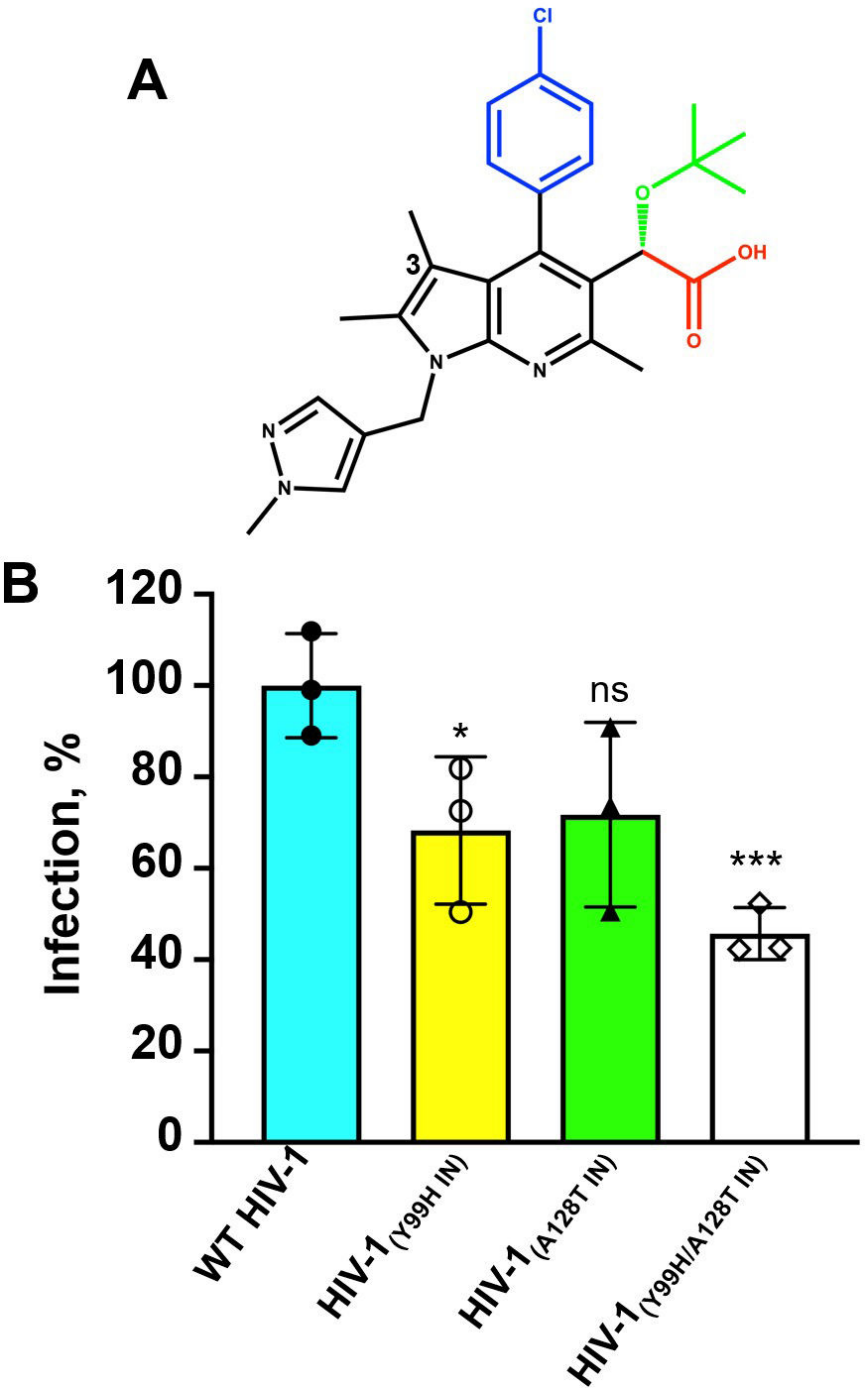
(**A**) The chemical structure of PIR. The separate functional groups are color-coded: carboxylate in red; *tert*-butoxyl in green; chlorophenyl in blue; and core pyrrolopyridine and methylpyrazole rings in black. The 3-methyl group on the pyrrolopyridine ring is indicated. (**B**) Infectivity of WT and indicated mutant viruses. The statistical significance was determined between WT and IN mutants by unpaired (two-tailed) *t*-test. For WT vs Y99H *P* = 0.0272; WT vs A128T *P* = 0.0611; WT vs Y99H/A128T *P* = 0.0008. *, *P* ≤ 0.05; ***, *P* ≤ 0.001; ns, not significant (*P* > 0.05).

Over the past decade, multiple ALLINI chemotypes with different core aromatic ring structures have been developed (1–8). Of these, pirmitegravir (PIR, Fig. 1A), which contains the core pyrrolopyridine ring, has recently advanced into phase 2a clinical trials. The cell culture-based viral breakthrough assays have identified IN mutations that arose under the selective pressure of PIR (5). Initial evolution of the HIV-1_(Y99H IN)_ phenotype was followed by the emergence of the HIV-1_(Y99H/A128T IN)_ variant at higher PIR concentrations. Here, we have investigated structural and mechanistic bases for the viral resistance to PIR. Surprisingly, we found that although Tyr99 and Ala128 are positioned within the V-shaped cavity at the CCD dimer, the Y99H/A128T IN changes did not substantially affect the direct binding of PIR to CCD. Instead, the steric hindrance induced by the resistant mutations prevented the CTD binding to the CCD_Y99H/A128T_ + PIR. By exploiting these structural findings, we have rationally developed an improved PIR analog EKC110, which exhibited ~14-fold higher potency against HIV-1_(Y99H/A128T IN)_ compared with its parental inhibitor.

## RESULTS

### Effects of Y99H, A128T, and Y99H/A128T IN mutations on antiviral activity of PIR

We evaluated how Y99H, A128T, and Y99H/A128T IN substitutions affect HIV-1 replication. The virus production from HEK293T cells transfected with full-length NL4.3 plasmids was not detectably influenced by these amino acid changes (Fig. S2). Infectivity of single mutant viruses HIV-1_(Y99H IN)_ and HIV-1_(A128T IN)_ in TZM-bl cells were reduced by ~30%, whereas HIV-1_(Y99H/A128T IN)_ was ~55% less infectious compared with WT HIV-1 (Fig. 1B). The antiviral assays performed with PIR revealed that HIV-1_(Y99H IN)_ conferred relatively modest (~4-fold) resistance to the inhibitor, whereas larger reductions in the inhibitor potency were observed with respect to HIV-1_A128T IN_ (~13-fold) and HIV-1_Y99H/A128T IN_ (>150-fold) compared with their WT counterpart (Table 1; Fig. S3).

**TABLE 1:** Antiviral activities of PIR against WT and indicated mutant viruses

HIV-1_NL4.3_	PIR EC_50_ (nM)
WT	10.4 ± 0.4
Y99H	39.9 ± 4.8
A128T	132.9 ± 31.9
Y99H/A128T	1,559.8 ± 62.2

### Biochemical mechanisms of the IN_Y99H/A128T_ resistance to PIR

Our biochemical assays have focused on elucidating the underlying mechanism for the major drug-resistant IN_Y99H/A128T_ protein. Size exclusion chromatography (SEC) experiments revealed that the predominant oligomeric form of both WT and Y99H/A128T INs was a tetramer (Fig. S4), which was previously shown to be the authentic antiviral target of ALLINIs (13).

**Fig 2:**
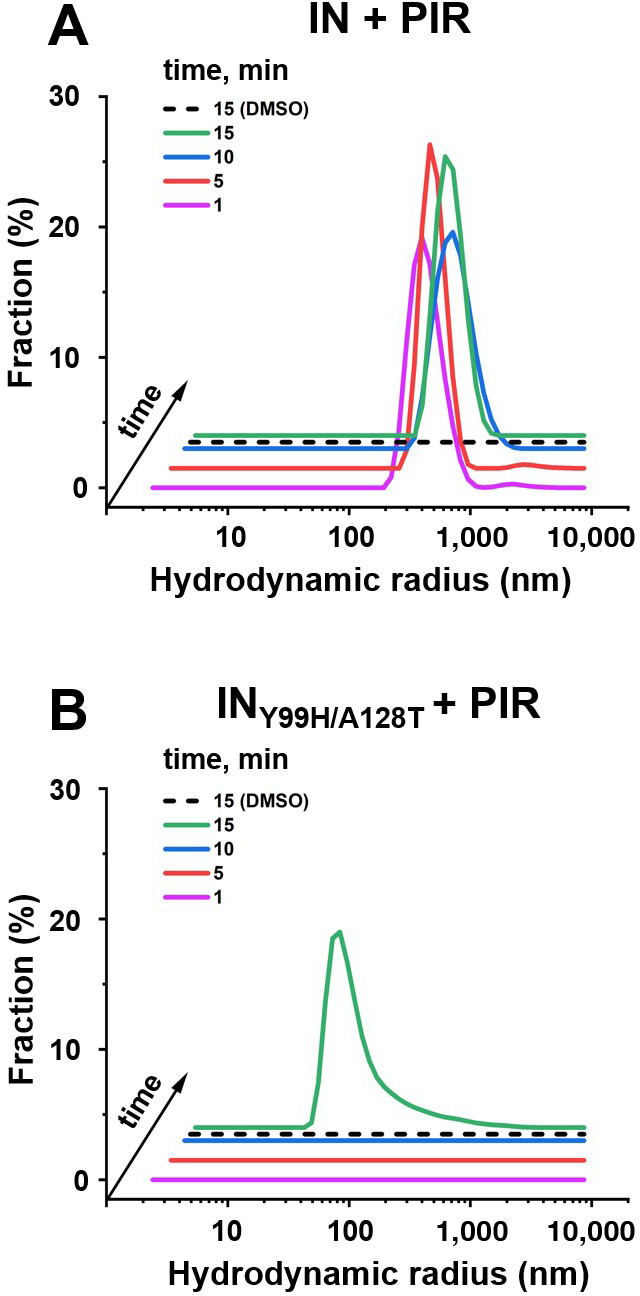
DLS analysis of PIR induced aberrant IN multimerization. Five hundred nanomolar PIR was added to 200 nM full-length WT IN (**A**) or IN_Y99H/A128T_, (**B**) and DLS signals were recorded at indicated times (1–15 min). DMSO controls are shown after incubation of full-length IN proteins for 15 min to indicate that these proteins remained fully soluble in the absence of PIR.

Published dynamic light scattering (DLS) studies demonstrated that the ALLINI-induced IN aggregation is a dynamic process and yields heterogeneous species (3). Here, we extended the application of DLS assays to study PIR-induced aggregation of full-length WT and Y99H/A128T INs. The inhibitor rapidly (within 1 min) induced hyper-multimerization of WT IN, yielding large particles with hydrodynamic radii of >400 nm (Fig. 2A). In contrast, no protein aggregation was observed after 10 min of PIR addition to IN_Y99H/A128T_ (Fig. 2B). Instead, initial inhibitor-induced hyper-multimerization of IN_Y99H/A128T_ with particle sizes of <100 nm was detected only after 15 min (Fig. 2B).

**Fig 3:**
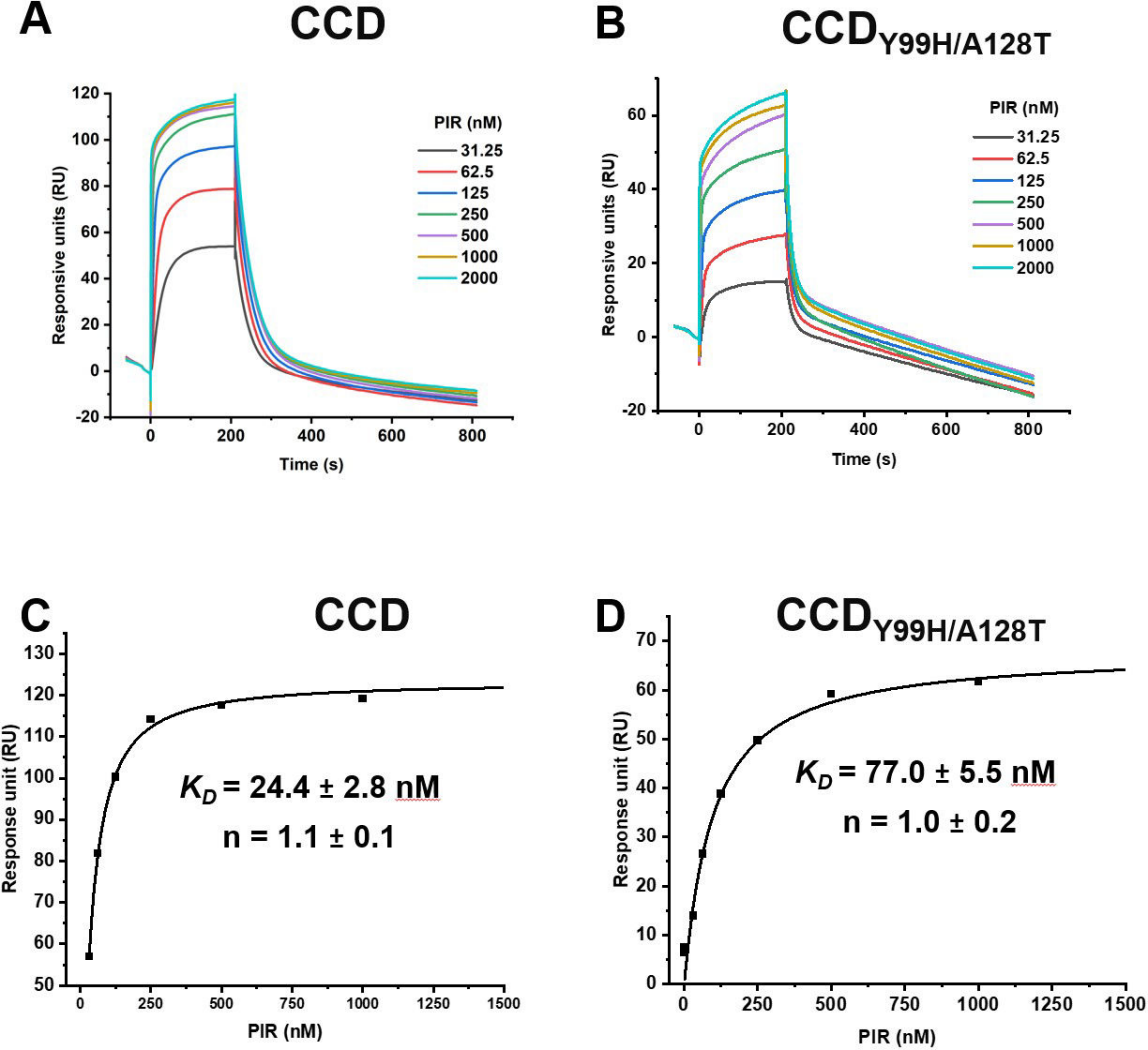
SPR analysis of PIR binding to WT CCD and CCD_Y99H/A128T_. Representative sensorgrams for PIR binding to WT CCD (**A**) vs CCD_Y99H/A128T_ (**B**). PIR concentrations are indicated. The dissociation constant (*K_D_*) and the Hill coefficient (n) for PIR + CCD (**C**) and PIR + CCD_Y99H/A128T_ (**D**) were determined using the Hill equation.

To understand how the Y99H/A128T IN mutations affect the inhibitor binding to the CCD dimer, we conducted surface plasmon resonance (SPR) assays. Surprisingly, we observed only modest differences between PIR binding to CCD_Y99H/A128T_ (*K_D_* of ~77 nM) vs WT CCD (*K_D_* of ~24 nM) (Fig. 3). Clearly, this ~3-fold change in the binding affinity does not explain the marked resistance (>150-fold) to PIR conferred by HIV-1_(Y99H/A128T IN)_ compared with the WT virus (Table 1).

**Fig 4:**
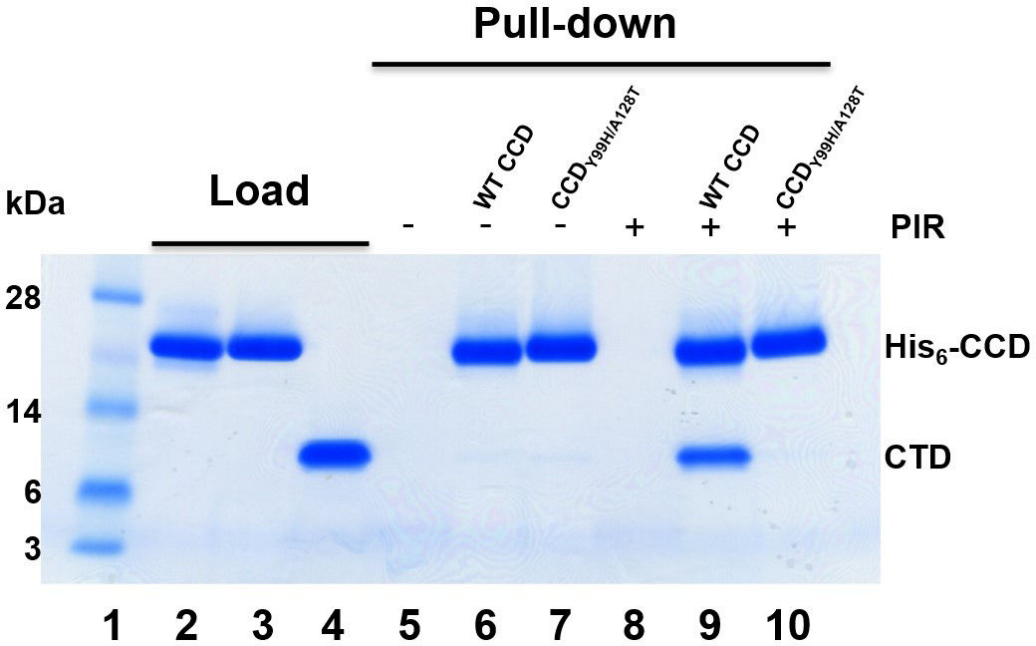
Affinity pull-down assays to probe PIR-induced CCD-CTD interactions. Lane 1: molecular weight markers; lanes 2–4: loads of His_6_-CCD (lane 2), His_6_-CCD_Y99H/A128T_ (lane 3), and tag-less CTD (lane 4); lanes 5–7: affinity pull-down using Ni beads of CTD alone (lane 5, control), His_6_-CCD + CTD (lane 6), His_6_-CCD_Y99H/A128T_ + CTD (lane 7) in the absence of PIR; and lanes 8–10: affinity pull-down using Ni beads of CTD + PIR (lane 8, control), His_6_-CCD + PIR + CTD (lane 9), His_6_-CCD_Y99H/A128T_ + PIR + CTD (lane 10).

Upon binding to the V-shaped cavity at the CCD dimer interface, ALLINIs act as molecular glues to recruit CTD (31–33) (also see Fig. S1C). Therefore, we tested how Y99H/A128T IN mutations affected the formation of the CCD-PIR-CTD complex. For this, we have developed an affinity pull-down assay to capture the CTD specifically bound to the His_6_-CCD in the complex with PIR. The results in Fig. 4 demonstrate that CTD was selectively pulled down by His_6_-CCD only in the presence, but not in the absence, of PIR (Fig. 4, compare lane 9 with 6). In sharp contrast to WT CCD, His_6_-CCD_Y99H/A128T_ failed to bind to CTD in the absence or presence of PIR (Fig. 4, lanes 7 and 10).

Taken together, our biochemical studies indicate that the Y99H/A128T IN changes do not substantially affect the direct binding of PIR to its cognate V-shaped cavity at the CCD dimer. Instead, the Y99H/A128T mutations strongly interfere with CTD binding to the CCD + PIR complex. Consequently, IN_Y99H/A128T_ confers the marked resistance with respect to the ability of PIR to induce aberrant IN multimerization.

### The structural basis for the IN_Y99H/A128T_ resistance to PIR

**Fig 5:**
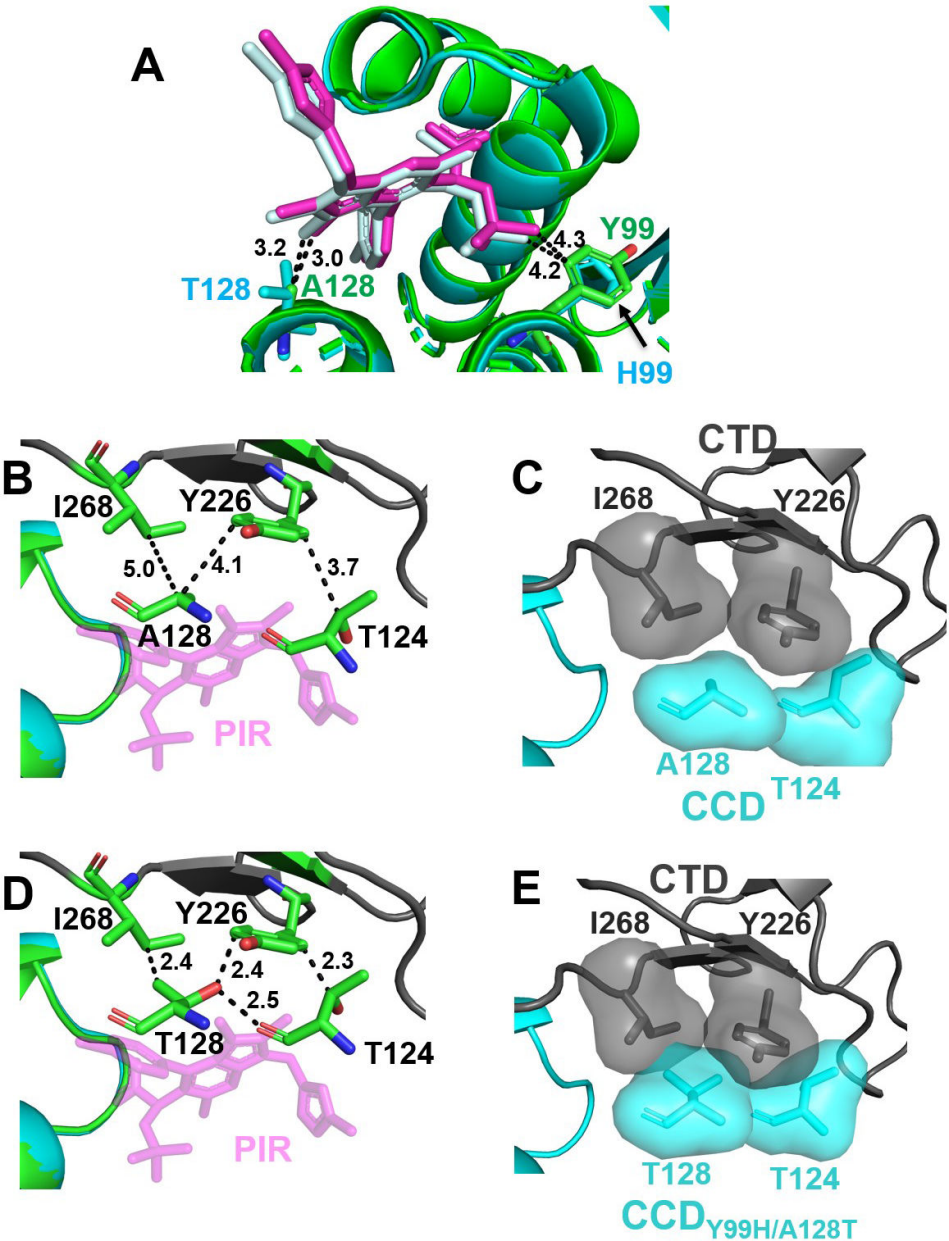
The structural analysis of PIR interactions with WT and drug-resistant proteins. (**A**) Superimposed crystal structures of WT CCD (green) + PIR (magenta) and CCD_Y99H/A128T_ (cyan) + PIR (pale cyan). The closest distances were measured from the 3-methyl group of PIR‘s pyrrolopyridine ring to C_β_ of Ala128 and Thr128, as well as from the methyl group on PIR’s *tert*-butoxy to Tyr99 and His99. (**B**) The closest distances between indicated CCD and CTD residues are shown in the structure of the WT CTD-CCD + PIR complex. Specifically, the distances from C_α_ of Ala128 to C4 of Ile268 and C3 of the aromatic ring of Tyr226, as well as from C_β_ of Thr124 to C6 of the aromatic ring of Tyr226 are indicated. (**C**) Van der Waals surface for indicated residues are shown in the structure of WT CTD-CCD + PIR. The inhibitor is not shown for clarity. (**D**) The closest distances between indicated CCD and CTD residues are shown when the structure of CCD_Y99H/A128T_ + PIR is superimposed onto the structure of WT CTD-CCD + PIR. Specifically, the distances from C_γ_ of Thr128 to C4 of Ile268, from the Thr128 side chain to C3 of the aromatic ring of Tyr226, and from C_β_ of Thr124 to C6 of the aromatic ring of Tyr226 are indicated. In addition, the hydrogen bond formed between Thr128 and Thr124 side chains is illustrated. (**E**) Van der Waals surface for indicated residues reveals steric clashes observed by overlapping, shaded surfaces when the structure of CCD_Y99H/A128T_ + PIR superimposed onto the structure of WT CTD-CCD + PIR. The inhibitor is not shown for clarity.

We have solved X-ray structures of PIR bound to both WT CCD and CCD_Y99H/A128T_ (Tables S1 and S2), which revealed very similar binding of the inhibitor to these proteins (Fig. 5A). Both the aromatic ring of Tyr99 and the imidazole ring of His99 adopt very similar positions as they extend inside the CCD-CCD dimer interface and away from the bound PIR (Fig. 5A). The Ala128 side chain is surface-exposed and extends toward the 3-methyl group of the core pyrrolopyridine ring system. However, the substitution of Ala128 with the bulkier and polar Thr128 did not seemingly alter the inhibitor positioning in the V-shaped pocket, and the distances from the 3-methyl group to the closest C_β_ of Ala128 and Thr128 were nearly identical (~3.0 Å vs 3.2 Å, Fig. 5A). Furthermore, the polar group of Thr128 points away from the inhibitor. Taken together, these structural findings agree well with our biochemical results, indicating that the Y99H/A128T changes do not substantially affect the functional oligomerization of full-length IN (Fig. S4) or the direct binding of the inhibitor to the CCD dimer (Fig. 3).

Recently, two-domain HIV-1 IN CTD-CCD constructs were developed to study ALLINI-induced CTD-CCD interactions (32). Our efforts to obtain a crystal structure for the CTD-CCD_Y99H/A128T_ + PIR complex have not been successful likely due to the inability of CTD to bind to the CCD_Y99H/A128T_ + PIR complex (Fig. 4). Therefore, to understand how Y99H/A128T mutations affect the CTD binding, we superimposed our crystal structure of CCD_Y99H/A128_ + PIR onto the recently reported structure of the CTD-CCD + PIR (32) (Fig. 5B and E). Figure 5B and C shows the relative positioning of CCD residues Thr124 and Ala128 with respect to CTD residues Tyr226 and Ile268 to indicate the lack of steric clashes at the WT CCD-PIR-CTD interface (32).

Of note, the change of Ala128 to the bulkier and polar Thr128 creates steric hindrance with respect to CTD residues Tyr226 and Ile268 (Fig. 5D and E). Additionally, Thr128 indirectly triggers yet another steric clash between CCD Thr124 and CTD Tyr226 (Fig. 5D and E). The root cause for this is a hydrogen bond formed between Thr128 and Thr124 side chains in our crystal structure of the CCD_Y99H/A128T_ + PIR complex (Fig. 5D), which in turn repositions Thr128 too close to CTD Tyr226 (compare the distances in Fig. 5B and D). These structural observations are consistent with the biochemical results, demonstrating that the CCD_Y99H/A128T_ + PIR complex does not effectively interact with the CTD (Fig. 4).

### The development of a PIR analog EKC110 with improved antiviral potency

**Fig 6:**
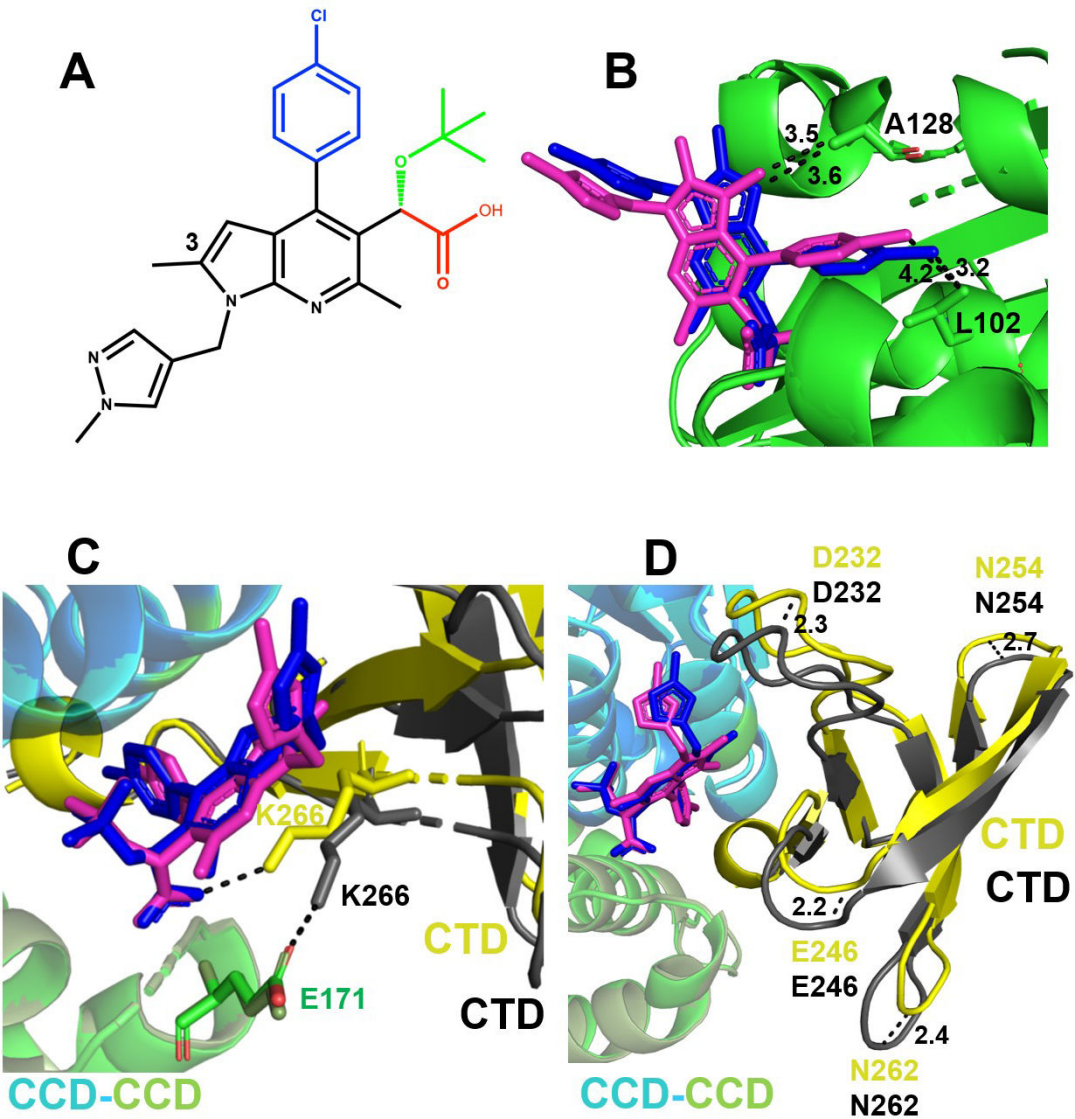
The structural analysis of EKC110 interactions with the CCD and the CTD-CCD. (**A**) The chemical structure of EKC110. The separate functional groups are color-coded: carboxylate in red; *tert*-butoxyl in green; chlorophenyl in blue; and core pyrrolopyridine and methylpyrazole rings in black. (**B**) The crystal structure of the CCD + EKC110 is superimposed onto the CCD + PIR, which reveals a noticeable tilt of the EKC110 pyrrolopyridine core toward A128 compared with PIR. The closest distances from C_β_ of Ala128 to C3 of the EKC110’s pyrrolopyridin ring and the 3-methyl substituent of PIR’s pyrrolopyridin ring are shown. In addition, the closest distances between C_γ_ of Leu102 and the chlorine atoms of PIR and EKC110 are indicated. (**C**) The crystal structure of the CTD-CCD + EKC110 is superimposed onto the CTD-CCD + PIR, which reveals that Lys266 side chain (gray) forms a salt bridge with the Glu171 side chain (green) in the presence of PIR (magenta), whereas Lys266 side chain (yellow) engages with the pharmacophore carboxylate of EKC110 (blue). (**D**) The crystal structure of the CTD-CCD + EKC110 is superimposed onto the CTD-CCD + PIR to show repositioning of the CTD in the presence of EKC110 vs PIR. CTDs are shown in yellow and gray in EKC110 + CTD CCD and PIR + CTD CCD structures, respectively. PIR and EKC110 are in magenta and blue. The distances between the C_α_ atoms for indicated residues (Asp232, Glu246, Asn254, and Asn262) in the EKC110 +CTD CCD vs the PIR + CTD CCD structures are shown.

From examining crystal structures of PIR bound to WT CCD and CCD_Y99H/A128T_ (Fig. 5A), we noticed that the 3-methyl group of the core pyrrolopyridine ring system extends toward both Ala128 and Thr128, and partly, limits PIR accessibility within the V-shaped pocket. We hypothesized that removing the 3-methyl group could enable a modified PIR analog to position itself deeper within the CCD dimer and potentially reduce steric hindrance with respect to the CTD binding to the CCD_Y99H/A128T_ + PIR complex. To test this notion, we have synthesized the PIR analog EKC110 lacking the 3-methyl group (Fig. 6A). Excitingly, EKC110 exhibited ~14-fold improved potency against HIV-1_(Y99H/A128T IN)_ compared with PIR (Table 2). Furthermore, EKC110 was ~2-fold more potent than PIR against WT HIV-1.

**TABLE 2:** Antiviral activities of EKC110 vs PIR

HIV-1_NL4.3_	PIR EC_50_ (nM)	EKC110 EC_50_ (nM)	Potency increase, fold
WT	10.4 ± 0.4	4.5 ± 0.2	~2.3
Y99H/A128T	1,559.8 ± 62.2	110.7 ± 5.2	~14
Resistance, fold	~150	~25	

### The structural basis for EKC110 interactions with WT and Y99H/A128T CCDs

We have solved the X-ray crystal structures of EKC110 bound to WT CCD, CCD_Y99H/A128T_, and WT CTD-CCD (Tables S1 and S2; Fig. 6; Fig. S5), whereas the CTD-CCD_Y99H/A128T_ + EKC110 complex did not yield crystals. A comparative analysis of EKC110 with PIR reveals both similarities and notable differences between these inhibitors (Fig. 6B through D; Fig. S5). In common with other members of the ALLINI class of inhibitors, the EKC110 key pharmacophore carboxylic acid establishes bidentate hydrogen bonding with backbone amides of Glu170 and His171 (Fig. S5). Additionally, the side chain of Thr174 hydrogen bonds with both EKC110 carboxylate and *tert*-butoxy moiety, which is crucial for the high potency of ALLINIs (Fig. S5).

We have observed the following significant differences between EKC110 and PIR binding to either the CCD or the CTD-CCD (Fig. 6B through D). EKC110 core pyrrolopyridine and methylpyrazole rings are slightly shifted compared with PIR. More specifically, because of the lack of the 3-methyl group, the EKC110 core pyrrolopyridine ring moves closer to and forms hydrophobic interactions with Ala128 (Fig. 6B). Consequently, EKC110 chlorobenzene group extends deeper inside the CCD-CCD dimer cavity toward Leu102 compared with its parental PIR (Fig. 6B).

Another significant change is seen for the CTD interaction with the CCD-PIR vs the CCD-EKC110 (Fig. 6C and D). Specifically, the CTD is anchored to the CCD-PIR interface by a salt bridge between CTD Lys266 and CCD Glu171. By contrast, in the CCD-EKC110-CTD complex, Lys266 establishes ionic interactions with the pharmacophore carboxylate of EKC110 (Fig. 6C). In addition, we note that although CCD dimers in the presence of PIR and EKC110 are superimposed closely, there was considerable repositioning of the CTD in the CCD-EKC110-CTD vs the CCD-PIR-CTD complex (Fig. 6D).

**Fig 7:**
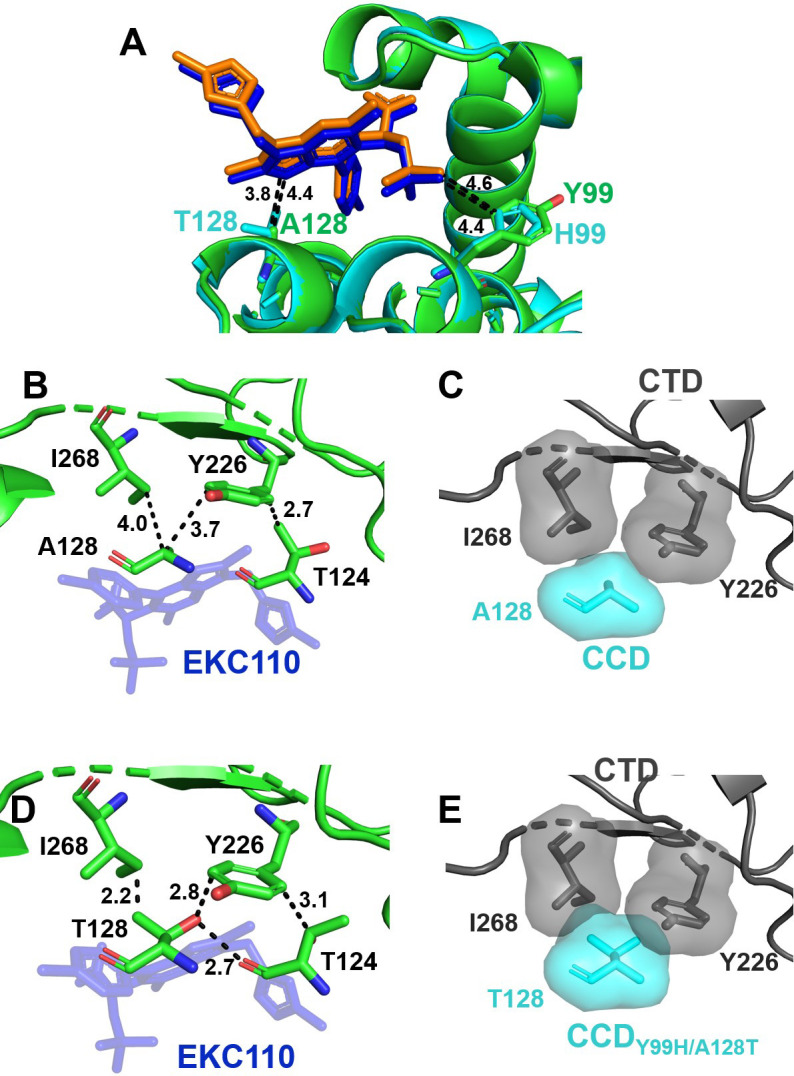
Structural analysis of EKC110 interactions WT and drug-resistance proteins. (**A**) Superimposed crystals structures of WT CCD (green) + EKC110 (blue) and CCD_Y99H/A128T_ (cyan) + EKC110 (orange). The closest distances from the EKC110’s pyrrolopyridine ring to C_β_ of Ala128 and Thr128, as well as from the methyl group on EKC110’s *tert*-butoxy to Tyr99 and His99 are indicated. (**B**) The closest distances between indicated CCD and CTD residues in the structure of WT CTD-CCD + EKC110 are shown. Specifically, the distances from C_α_ of Ala128 to C4 of Ile268 and C3 of the aromatic ring of Tyr226 as well as from C_γ_ of Thr124 to C6 of the aromatic ring of Tyr226 are indicated. (**C**) Van der Waals surface for indicated residues are shown in the structure of WT CTD-CCD + PIR. Thr124, which does not encounter any steric hindrance, and the inhibitors are not shown for clarity. (**D**) The closest distances between indicated CCD and CTD residues are shown when the structure of CCD_Y99H/A128T_ + EKC-110 is superimposed onto the structure of WT CTD-CCD + EKC-110. Specifically, the distances from C_γ_ of Thr128 to C4 of Ile268, from the side chain of Thr128 to C3 of the aromatic ring of Tyr226, and from C_β_ of Thr124 to the C6 of the aromatic ring of Tyr226 are indicated. In addition, the hydrogen bond between the side chains of Thr128 and Thr124 is illustrated. (**E**) Van der Waals surface for indicated residues reveals steric clashes observed by overlapping, shaded surfaces when the structure of CCD_Y99H/A128T_ + EKC110 superimposed onto the structure of WT CTD-CCD + EKC110. Thr124, which does not encounter any steric hindrance, and the inhibitors are not shown for clarity.

The comparative analysis of the crystal structures of EKC110 bound to CCD vs CCD_Y99H/A128T_ revealed that the drug-resistant substitutions did not detectably influence the inhibitor binding to the V-shaped cavity (Fig. 7A). Since the CTD-CCD_Y99H/A128T_ + EKC110 complex was not amenable to X-ray crystallography, we superimposed the crystal structure of CCD_Y99H/A128T_ + EKC110 onto the CCD-EKC110-CTD structure (Fig. 7B through E). Unlike the CCD-EKC110-CTD complex, which did not exhibit any steric clashes (Fig. 7B and C), the drug-resistant A128T change induced steric hindrance with respect to both Tyr226 and Ilu268 (Fig. 7D and E) These observations with EKC110 (Fig. 7D and E) are similar to those with PIR (Fig. 5D and E).

However, unlike the CCD_Y99H/A128T_ + PIR + CTD interface, where the additional steric hindrance is seen between Thr124 and Tyr226 (Fig. 5D and E), Thr124 is sufficiently distant from Tyr226 to avoid steric conflicts in the CCD_Y99H/A128T_ + EKC110 + CTD complex (Fig. 7D). Note, although similarly to the CCD_Y99H/A128T_ + PIR structure (Fig. 5D), Thr124 and Thr128 side chains form a hydrogen bond in the CCD_Y99H/A128T_ + EKC110 complex (Fig. 7D) because of the repositioning of the CTD in the presence of EKC110 (Fig. 6C), Thr124 can avoid the steric conflict with Tyr226.

In summary, a comparative analysis of the two inhibitors reveals that the drug-resistance mutations induce multiple steric clashes at the CCD_Y99H/A128T_ + PIR + CTD interface; EKC110 partly rather than fully evades these conflicts. These structural findings agree with the virology results (Table 2), demonstrating that although Y99H/A128T mutations still confer ~25-fold resistance to EKC110 compared with WT HIV-1 (Table 2), EKC110 exhibits ~14-fold improved potency compared to PIR against HIV-1_(Y99H/A128T IN)_.

### MD simulations and energetics of PIR and EKC110 interactions with WT and Y99H/A128T CCDs

**Fig 8:**
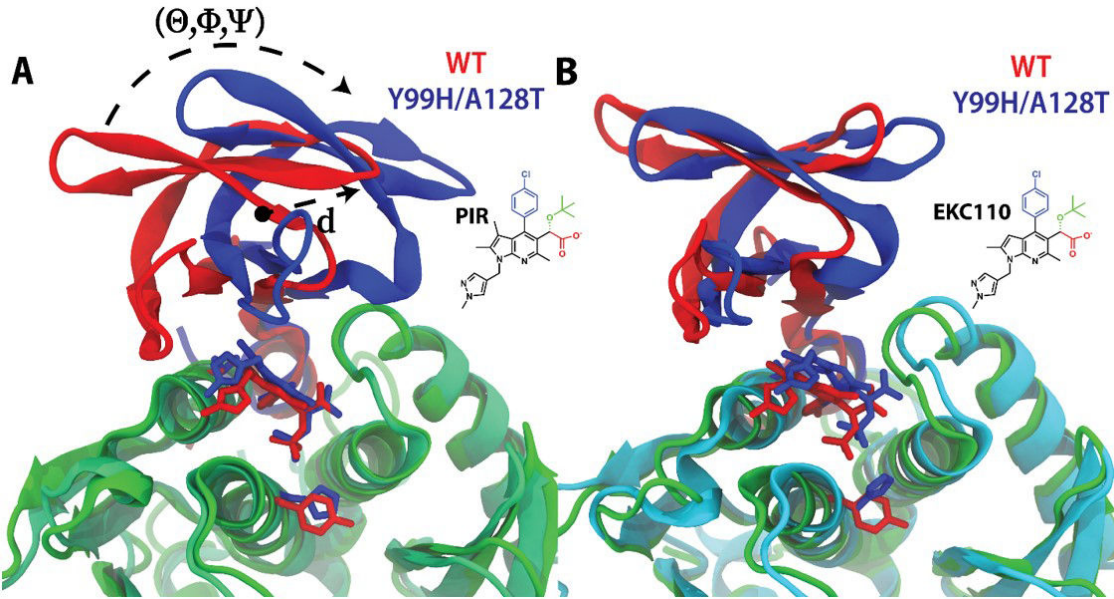
MD simulation of the CTD interactions with WT CCD vs CCD_Y99H/A128T_ in the complex with PIR (**A**) and EKC110 (**B**). Displacement of the CTD domain from the PIR + CCD_Y99H/A128T_ complex is measured with a center-of-mass displacement of d = 4.85 Å and rotations along the principal axes of inertia of θ = 26.66°, Φ = 23.84°, and Ψ = 11.81°. WT CCD and CCD_Y99H/A128T_ are colored cyan and green, respectively. CTDs interacting with WT CCD and CCD_Y99H/A128T_ are colored red and blue, respectively.

To quantify the effect of the Y99H/A128T IN mutations on the binding of PIR and EKC110 at the CCD-CTD interface, we performed 1 μs MD simulations (Fig. 8; Fig. S6). Both ALLINIs greatly stabilized interactions between WT CCD and CTD. The average root mean squared deviations (RMSD) were 3.0 ± 0.3 Å and 3.0 ± 0.3 Å for the CCD-PIR-CTD complex; 2.9 ± 0.2 Å and 3.0 ± 0.3 Å for the CCD-EKC110-CTD complex; and 3.2 ± 0.4 Å and 6.6 ± 1.2 Å for apo CCD and CTD in the absence of ALLINIs. Of note, during 1 μs MD simulations, the CTD domain separated from apo CCD, whereas the CCD-ALLINI-CTD interface remained stable (Fig. S6A) and exhibited an overall decrease in the RMSF across all IN residues (Fig. S6B).

Although our analysis revealed a gradual displacement of CTD from the CCD_Y99H/A128T_ + PIR complex compared with the WT CCD-PIR-CTD complex (Fig. 8A; Fig. S6; Movie S1), the CTD displacement was significantly reduced in the context of the CCD_Y99H/A128T_-EKC110-CTD complex compared with its WT CCD-EKC110-CTD counterpart (Fig. 8B; Fig. S6). Although the CTD was slightly displaced from CCD_Y99H/A128T_-EKC110-CTD throughout the simulation compared with the WT CCD-EKC110-CTD complex, these shifts in orientation were transient and the CTD domain returned to its WT-like orientation in the presence of EKC110 but not in the presence of PIR (Fig. S6).

To further characterize the CTD displacement, we measured the internal volume of the CCD-CTD binding pocket throughout the simulations (Fig. S7). For the Y99H/A128T IN in complex with PIR, the CCD-CTD binding pocket exhibited an initial volume of 375 Å^3^, which increased to 433 Å^3^ by the end of the simulation. This volume increase is correlated to the displacement of the CTD domain from the CCD_Y99H/A128T_-PIR-CTD complex compared with the WT CCD-PIR-CTD structure. Throughout 1 μs MD simulations, the WT CCD-PIR-CTD complex exhibited an average interface volume of 377 Å^3^ with an uncertainty of 49 Å^3^, whereas the average volume of the CCD_Y99H/A128T_-PIR-CTD interface increased to 397 Å^3^ with a uncertainty of 54 Å^3^. A Student *t*-test comparison of the volume distributions yielded a *P*-value of 3.79 × 10^−211^, indicating that there is a statistically significant difference between the two volumes.

The CCD_Y99H/A128T_-EKC110-CTD complex exhibited an initial volume of 375 Å^3^ and after 1 μs MD simulations, the volume was largely unchanged (372 Å^3^), which is consistent with the more stable nature of the CCD-EKC110-CTD interface (32). For the CCD_Y99H/A128T_-EKC110-CTD system, we measured an average volume of 372 Å^3^ and a standard deviation of 58 Å^3^, which were reduced from the average volume of 391 Å^3^ and a standard deviation of 54 Å^3^ measured from the WT CCD-EKC110-CTD complex. The Student *t*-test revealed a *P*-value of 1.42 × 10^−119^, indicating a statistically significant difference between the volume distributions. This reduction in volume can be attributed to the Y99H substitution, as replacing a Tyr with the less bulky His allows α-helix 1 from one CCD subunit and α-helix 5 from the partner CCD subunit to be packed closer together.

Taken together, the MD simulation results suggest that Y99H/A128T mutations lead to changes in observed volumes of the ALLINI binding pocket (6) at the CCD-ALLINI-CTD interface and more readily displace CTD from the CCD_Y99H/A128T_ + PIR than the CCD_Y99H/A128T_ + EKC110 complex.

### FEP calculations

To quantify how Y99H, A128T, and Y99H/A128T mutations affected CCD-ALLINI-CTD interactions, we performed FEP calculations (Fig. S8). For this, we computed the relative free energy difference (ΔΔ*G*) between the WT CCD vs CCD containing Y99H, A128T, and Y99H/A128T mutations for their ability to form the CCD-ALLINI-CTD complexes (Fig. S8). The single Y99H mutation carried a similar energetic penalty for both CCD-PIR-CTD (ΔΔ*G* = 0.7 ± 0.1 kcal/mol) and CCD-EKC110-CTD (ΔΔ*G* = 1.0 ± 0.9 kcal/mol) complexes, whereas the single A128T mutation yielded a much higher ΔΔ*G* for the CCD-PIR-CTD complex (ΔΔ*G* = 5.2 ± 0.5 kcal/mol) compared with the free energy differences seen for the CCD-EKC-CTD complex (ΔΔ*G* = 2.1 ± 0.2 kcal/mol). The drug-resistant mutations induced a free energy change of ΔΔ*G* = 7.3 ± 1.0 kcal/mol for the CCD_Y99H/A128T_-PIR-CTD complex, which was higher than the free energy change of ΔΔ*G* = 5.1 ± 0.7 kcal/mol measured for the CCD_Y99H/A128T_-EKC110-CTD complex. These findings suggest that the Y99H/A128T mutations are more unfavorable for PIR than EKC110.

Collectively, MD simulation results and FEP calculations indicate that Y99H/A128T mutations adversely affect both PIR and EKC110 albeit the effects of the drug-resistance mutations on PIR are more pronounced than EKC110. These findings agree with our virology data, which show that although Y99H/A128T mutations confer resistance to both PIR and EKC110 (Table 2), EKC110 exhibits ~14-fold improved potency compared with PIR against HIV-1_(Y99H/A128T IN)_.

### Biochemical characterization of EKC110 interactions with WT and Y99H/A128T INs

**Fig 9:**
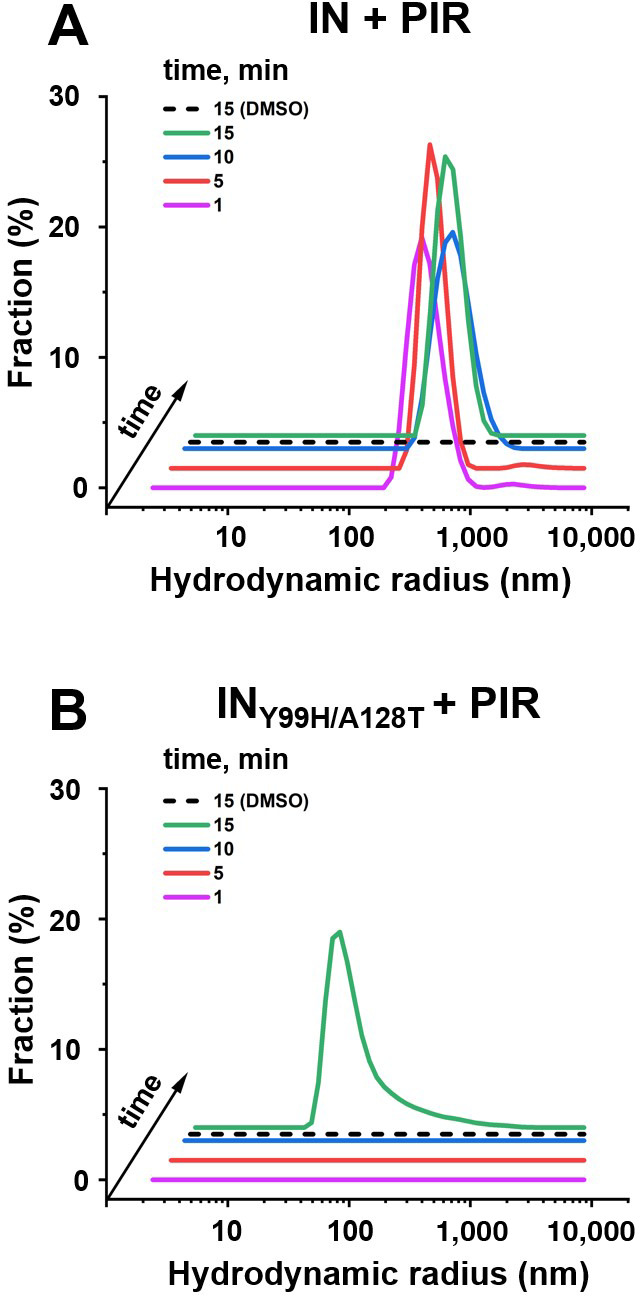
Interactions of EKC110 with full-length HIV-1 IN. DLS analysis of EKC110 induced aberrant IN multimerization. Five hundred nanomolar EKC110 was added to 200 nM full-length WT IN (**A**) or IN_Y99H/A128T_, (**B**) and DLS signals were recorded at indicated times (1–15 min). DMSO controls are shown after incubation of full-length IN proteins for 15 min to indicate that these proteins remained fully soluble in the absence of EKC110.

DLS assays with full-length proteins revealed that unlike PIR, EKC110 effectively induced aberrant multimerization of both WT and Y99H/A128T INs (compare Fig. 9 and 2). These findings are consistent with the virology assays in Table 1 demonstrating that EKC110 is ~14-fold more potent than PIR against HIV-1_(Y99H/A128T IN)_.

Next, we characterized interactions of EKC110 with isolated IN domains (Fig. S9 and S10). SPR experiments in Fig. 9 demonstrate that EKC110 is bound to the CCD and the CCD_Y99H/A128T_ with *K_D_* values of ~29 nM and ~140 nM, respectively. Modestly (~5-fold) reduced binding affinity of EKC110 with respect to the drug-resistance CCD_Y99H/A128T_ compared with its WT counterpart does not fully account for more substantial (~25-fold) resistance observed in virology assays (Table 2).

The pull-down assays in Fig. S10 show that the CCD_Y99H/A128T_ + EKC110 exhibited marked resistance with respect to the CTD. These results are consistent with structural findings (Fig. 7D and E), indicating that EKC110 partly rather than fully evades multiple steric clashes observed at the CCD_Y99H/A128T_ + PIR + CTD interface. We also note that our biochemical experiments with full-length INs (Fig. 9), but not with isolated CCD and CTD (Fig. S10), were able to delineate an improved potency of EKC110 compared with PIR against Y99H/A128T IN. Below, we discuss how the CCD-ALLINI-CTD interactions provide a crucial albeit incomplete understanding of the inhibitor-induced aberrant aggregation of full-length IN and the drug resistance to ALLINIs.

## DISCUSSION

Our multidisciplinary studies have elucidated an unexpected mechanism of the viral resistance to PIR. Although both Tyr99 and Ala128 are located within the V-shaped cavity at the CCD dimer, the Y99H/A128T mutations did not substantially affect direct binding of PIR to the CCD dimer or functional oligomerization of the full-length IN. Instead, these drug-resistant mutations introduced steric hindrance at the PIR-mediated CCD-CTD interface and impaired the ability of the CCD_Y99H/A128T_ + PIR complex to bind CTD. Consequently, full-length IN_Y99H/A128T_ was substantially more resistant to the PIR-induced hyper-multimerization than its WT counterpart. PIR was >150-fold less potent against HIV-1_(Y99H/A128T IN)_ vs the WT virus.

Cell culture-based viral breakthrough assays with different ALLINIs consistently identified various drug resistance mutations in the vicinity of the inhibitors’ binding site on the CCD (1, 3, 5, 7, 8, 14, 29, 34, 35). By contrast, no mutations were detected within the CTD. These findings suggest that HIV-1 is more tolerant to the drug-resistant mutations within the V-shaped cavity at the CCD dimer interface than at the complementary CTD interface, which is composed of the invariant residues (32). Indeed, the Y99H/A128T IN changes only partly (~2-fold) reduced HIV-1 infectivity (Fig. 1B), whereas the mutations of the key CTD residues that engage with the CCD-ALLINI complex are detrimental for the virus (36, 37).

The A128T change is the most frequently detected resistance mutation against different ALLINI chemotypes (1, 5, 6, 14, 34). Previous mechanistic studies with this IN mutation helped delineate that the primary mode of action of ALLINIs was through inducing hyper-multimerization of IN rather than inhibiting IN binding to LEDGF/p75 (14). Indeed, the A128T change did not detectably affect the ALLINI potency for inhibiting IN-LEDGF/p75 binding. Instead, IN_A128T_ was substantially more resistant to the inhibitor-induced hyper-multimerization than WT IN (14). However, the previous structural studies were limited to the ALLINI-CCD interactions, and the underlying mechanism for the A128T IN resistance remained obscure (14). Our studies here reveal the importance of the ALLINI-induced CCD-CTD interface for the emergence of the Y99H/A128T IN-resistant viral phenotype. In turn, these findings raise a possibility that a number of previously reported resistant mutations that arise in response to different ALLINI chemotypes could also affect the inhibitor-induced CCD-CTD interactions.

Although the recent reports (6, 32, 33) and the structural studies conducted here have uncovered atomic details for the CCD-ALLINI-CTD interactions, we also note the following limitations of using isolated IN domains. These protein constructs lacked the NTD, which is required for functional tetramerization of full-length, unliganded IN (13). In turn, IN tetramers are the authentic antiviral target for ALLINIs (13). The deletion of the NTD or substitutions in the NTD, which compromised IN tetramerization markedly diminished the ability of ALLINIs to induce protein aggregation (13). Consistent with the previous observations (13), micromolar concentrations of isolated protein domains and inhibitors were needed to detect interactions between the CCD, ALLINIs, and the CTD (Fig. 4; Fig. S10). These relatively weak CCD-ALLINI-CTD interactions are highly sensitive to any steric hindrance introduced by drug-resistant mutations (Fig. 7D and E) and cannot delineate differential levels of resistance to PIR vs EKC110. By contrast, nanomolar concentrations of the full-length WT IN and ALLINIs were sufficient to induce effective protein aggregation (Fig. 2 and 8). Furthermore, the kinetics of the inhibitor-induced hyper-multimerization of full-length INs demonstrated differential effects of drug-resistant Y99H/A128T mutations with respect to PIR vs EKC110 (Fig. 2 and 9).

The structural studies to elucidate how the NTD contributes to potent ALLINI-induced aggregation of the full-length WT IN have not been fruitful thus far. We speculate that the NTD indirectly contributes to the mode of action of ALLINIs by markedly facilitating IN-IN interactions. Despite our current lack of understanding of the role of the NTD, our structural studies focused on CCD-ALLINI and CCD-ALLINI-CTD interactions provided us with a powerful means to rationally develop a PIR analog EKC110 with improved antiviral potency. EKC110 was more potent against full-length IN_Y99H/A128T_ *in vitro* and HIV-1_(Y99H/A128T IN)_ in infected cells compared with the parental PIR. These exciting results inform future efforts to develop second-generation ALLINIs with an enhanced barrier for resistance to their potential clinical use.

## MATERIALS AND METHODS

### Cell lines, virus infectivity, and antiviral assays

HEK293T (ATCC) and HeLa TZM-bl (NIH AIDS Reference and Reagent Program) cells were cultured in Dulbecco’s modified eagle medium (DMEM, Gibco) supplemented with 10% fetal bovine serum (FBS, Sigma–Aldrich) and 1% penicillin–streptomycin (PS, Gibco). Cells were maintained in the incubator at 37°C and 5% CO_2_. Cell lines used in this study were tested monthly for Mycoplasma contamination.

For virus infectivity assay, HEK293T cells (2–4 × 10^5^ cells/well in 6-well plates) were seeded 1 day prior to transfection of 2 µg full-length pNL4.3 plasmid containing WT or mutant INs using HilyMax transfection reagent (Dojindo Molecular Technologies, Inc.) in 1:3 ratio. The medium was replaced with fresh medium at 12–16 h post-transfection and incubated at 37°C. Forty-eight hours post-transfection, virus containing supernatants were collected, clarified, and filtered through 0.45 µm filter, and the levels of p24 were monitored by western blot. We used p24 normalized filtered viral supernatant from 293T cells to infect TZM-bl cells, which were seeded at 50,000 cells/well in 24-well plate. After incubation at 37°C for 3–4 h, the medium was removed and replaced with a fresh medium. The cells were collected at 48 h post-infection, and virus infectivity was measured by luciferase assay (Promega).

To determine the antiviral potency of ALLINIs, full replication cycle experiments were performed with HIV-1_NL4.3_ containing WT or mutant INs as described (3, 38). HEK293T cells (producer cells, 2–4 × 10^5^ cells/well in 6-well plate) were seeded 24 h before transfection. We transfected a 2-µg full-length pNL4-3 plasmid containing WT or mutant INs using HilyMax transfection reagent in 1:3 ratio. Four to 6 hours post-transfection, the transfection medium was changed with the medium containing indicated concentrations of PIR and EKC110 or the DMSO control. Forty-eight hours post-transfection, virus containing supernatants were collected, clarified, and filtered through 0.45 µm filter. TZM-bl cells (target cells) were seeded 24 h prior to infection (~50,000 cells/well in 24-well plates). The inhibitors were added to the medium at concentrations matching those in producer cells. Subsequently, 50 µL virus containing supernatants were added to the cells. The cells were incubated for 3–4 h at 37°C, and the medium was removed and replaced with a fresh medium containing the same concentrations of inhibitors. Forty-eight hours post-infection, the cells were collected, and infectivity was measured using luciferase assay (Promega). Effective concentration (EC_50_) of the inhibitors was calculated using Origin software (OriginLab, Inc.). All virus infections were performed in the presence of 8 µg/mL polybrene, and values were expressed as mean ± standard deviation (SD). The statistical significance of virus infectivity of WT and integrase mutants was calculated by unpaired (two-tailed) *t*-test using the GraphPad Calculator.

### Synthesis of ALLINIs

PIR was synthesized as described (5). EKC110 (compound **19**) was prepared following the synthetic procedure outlined in scheme **1** (see Supplemental materials). Intermediate **2** was obtained by reacting commercially available diethyl malonate (**1**) and acetonitrile in presence of tin(IV) chloride (SnCl_4_) (39), whereas intermediate **6** was obtained by bromination of (1-methyl-1*H*-pyrazole-4-yl) methanol (**3**) (40) in the presence of 33% HBr in acetic acid followed by reaction with 5-methyl-2-pyrrolidinone (**5**) in the presence of NaH. Coupling of compounds **2** and **6** in the presence of POCl_3_ and subsequent cyclization in the presence of NaOEt afforded compound **8** (41). Oxidation of compound **8** with DDQ in toluene gave aromatized product **9,** which was converted to the corresponding triflate **10** by reaction with triflic anhydride (Tf_2_O) in the presence of triethylamine. Subsequent palladium-mediated Suzuki coupling with 4-chlorophenylboronic acid in the presence of potassium carbonate and Pd(PPh_3_)_4_ gave intermediate **11**. Aldehyde derivative **13** was obtained by first, reduction of the ethyl ester to the corresponding alcohol with DIBAL-H followed by oxidation with pyridinium chlorochromate. Reaction of aldehyde **13** with trimethylsilyl cyanide in the presence of ZnI_2_ gave silylated cyanohydrin **14** which, after hydrolysis with H_2_SO_4_ in methanol, provided hydroxyester **15**. Oxidation of the hydroxyl group with DMP followed by asymmetric reduction, using Corey-Bakshi-Shibata reagent ((*R*)-Me-CBS borane), gave the chiral alcohol **17**.^3^ Alkylation of **17** in the presence of *t*-butyl acetate and perchloric acid and further saponification of intermediate **18** led to target compound **19 (EKC110**).

### Recombinant proteins

Y99H, A128T, and Y99H/A128T mutations were introduced into the IN coding region in the context of the pET-15b vector using QuikChange XL Site-directed mutagenesis KIT (Agilent) and the manufacturer’s protocol. The DNA was miniprepped from DH10B cells, and the introduced mutations were verified by Sanger sequencing (Quintara Bioscience). All proteins (WT IN, IN_Y99H/A128T_, His_6_-CCD, His_6_-CCD_Y99H/A128T_, CTD, CTD-CCD, and CTD-CCD_Y99H/A128T_) were expressed in BL21 (DE3) cells and purified as described (11, 32). Purified proteins were examined using NuPAGE Bis-Tris 4-12% acrylamide gels with MES as the running buffer (Invitrogen). Proteins were stained AcquaStain Protein Gel Stain (Bulldog-Bio).

### Analytical SEC

Recombinant WT and mutant IN proteins were analyzed using Superdex 200 10/300 Gl column (GE Healthcare) with the running buffer containing 20 mM HEPES (pH 7.5), 1 M NaCl, 10% glycerol, and 5 mM BME at 0.3 mL/min flow rate. The protein stocks were diluted to 20 µM IN with the running buffer and incubated for 1 h at 4°C followed by centrifugation at 10,000 × *g* for 10 min. To estimate the multimeric state of IN proteins, we used the following standards: bovine thyroglobulin (670,000 Da), bovine gamma-globulin (158,000 Da), chicken ovalbumin (44,000 Da), horse myoglobin (17,000 Da), and vitamin B12 (1,350 Da). Retention volumes for different oligomeric forms of IN were as follows: tetramer ~12.5 mL, dimer ~14 mL, and monomer ~15–16 mL.

### SPR

The SPR biosensor binding experiments were performed using the Biacore T200 (Cytiva). A nitrilotriacetic acid (NTA) sensor chip was conditioned with 350 mM NiSO_4_ at a flow rate of 30 µL/min for 1 min. His_6_-CCD and His_6_-CCD_Y99H/128T_ proteins containing C-terminal hexa-His-tag were immobilized on the NTA sensor chip to about 2,000 response units. The running buffer contained 0.01 M HEPES (pH 7.4), 0.15 M NaCl, 0.05% vol/vol Surfactant P20 (Cytiva), and 5% DMSO. The desired concentrations of inhibitors were prepared by serially diluting the compounds in 100% DMSO and then by adding the running buffer (without DMSO) to reach a final DMSO concentration of 5%. The sensor chip was regenerated with 350 mM EDTA. For each interaction, background binding and drift were subtracted via a NTA reference surface. The sensorgrams were plotted using Origin software. Data were analyzed and fitted with a 1:1 kinetic model using the Origin software and the Hill equation:


$$y=V_{max}\times \;\frac{x^{n}}{k^{n}+\;x^{n}}$$

where *x* is the concentration of drug, *n* is the Hill coefficient, *k* is the apparent dissociation constant, and *V*_max_ is the highest reaction rate at saturating drug concentrations.

### DLS

The DLS assays were performed on a Malvern Zetasizer Nano s90 as described (3). Two hundred nanomolar full-length WT and Y99H/A128T INs were analyzed in the presence of 500 nM PIR or EKC110. These concentrations of the proteins and inhibitors were selected from preliminary experiments to allow us to monitor the kinetics of ALLINI-induced protein aggregation. For example, higher concentrations of IN and ALLINIs induced very rapid (<1 min) aggregation of the protein. Kinetic analysis was carried out at specified time points. In short, the reactions were performed in the DLS buffer (1 M NaCl, 2 mM MgCl_2_, 2 mM DTT, 50 mM HEPES, pH 7.5), which was filtered twice using 0.2 µm filter. One hundred micromolar stock solutions of ALLINIs were prepared in filtered DMSO and then added to the final concentration of 500 nM PIR or EKC110 in the 40 µL DLS reaction buffer containing 200 nM IN. Size distributions of the mixture were recorded at 1, 5, 10, and 15 min. For a negative control, the same amount of IN was mixed with 0.2 µL filtered DMSO (100%).

### CTD binding to the CCD + ALLINI complex

In total, 1.5 µM His_6_-CCD and His_6_-CCD_Y99H/A128T_ proteins were immobilized on Ni-NTA resin in the binding buffer containing 50 mM HEPES (pH 7.5), 200 mM NaCl, 2 mM MgCl_2_, 35 mM imidazole, 0.1% (vol/vol) Nonidet P40, and 0.1% BSA. Subsequently, 4.5 µM CTD was added in the presence of 3 µM ALLINI or the DMSO control. The mixtures were rotated for 30 min using Tube Revolver Rotator at a speed of 20 rpm for 30 min at RT. The resins were washed three times with the binding buffer to remove unbound proteins. The bound proteins were separated by SDS–PAGE electrophoresis and visualized by staining with Coomassie-Blue-like AcquaStain (Bulldog-Bio©).

### X-ray crystallography

The CCD and CCD_Y99H/A128T_ proteins were concentrated to 5 mg/mL and crystallized at 4°C using the hanging drop vapor diffusion method as described previously (42). Two microliters of protein were mixed with 2 µL reservoir, with 500 µL reservoir solution in the well, which contained 0.1 M (NH_4_)_2_SO_4_, 0.1 M sodium cacodylate (pH = 6.5), 10% PEG 8000, and 5 mM DTT. The cubic-shaped crystals reached 0.1–0.2 mm after 1–2 weeks. The soaking buffer was prepared the same as the mother liquid but supplemented with a 30% mixture of ethylene glycol, DMSO, and glycerol (1:1:1). The CCD and CCD_Y99H/A128T_ were soaked with either PIR or EKC110 (0.28 mM) in this cryoprotectant solution overnight before being flash-frozen in liquid nitrogen. Diffraction data were collected at 100 K by a Rigaku Micromax 007 with a Pilatus 200K 2D area detector at the University of Colorado Anschutz Medical Campus X-Ray Crystallography Facility.

For the CTD-CCD + EKC110 crystal structure, we used a 10 nM stock of EKC110 in DMSO. To prepare the protein-drug complexes, the CTD-CCD construct contained solubilizing F185K/W243E IN mutations as described (32). The protein was diluted to 0.6 mg/mL by buffer containing 20 mM Tris-HCl pH 7.5, 0.5 M NaCl, and then supplemented with 25 µM EKC110 in the presence of 5% (vol/vol) glycerol. Following incubation on ice for 10 min, the complexes were concentrated to 5 mg/mL using a 10 kDa cutoff VivaSpin device (Satorius). The crystals grew at room temperature (23°C) by adding 1 µL protein with 1 µL of reservoir containing 30 mM magnesium chloride, 30 mM calcium chloride, and 0.1 M imidazole-MES (Morpheus buffer system 1; Molecular Dimensions product code MD2-100-100), pH 6.5, 10% (wt/vol) PEG 8000, and 20% ethylene glycol. Crystals cryoprotected in the mother liquor supplemented with 30% glycerol were frozen by plunging them into liquid nitrogen.

### Structural studies

Data integration and reduction were performed with XDS (43). Molecular Replacement software Phaser (44) in the phenix (45) package was employed to solve all protein and ligand structures. Coot (46) and phenix.refine were used afterward to refine structures. TLS (47) and restraint refinement were done for the last step of structure refinement.

The CCD + ALLINI crystals belonged to space group P3_1_21 with cell dimensions: a = b = 72.09 and c = 65.91 Å with a 18.84 KDa monomer in the asymmetric unit. The structures were refined to approximately 1.9–2.1 Å with R_work_ = 0.22–0.26 and R_free_ = 0.26–0.30. PDB entry 6NUJ was used as the starting model, and CCD structures in complexes with PIR and EKC110 were deposited on PDB with codes 8D3S and 8S9Q, respectively. CCD_Y99H/A128T_ complexed with PIR and EKC110 were deposited on PDB with codes 8T52 and 8T5A, respectively.

The CTD-CCD + EKC110 crystals belonged to space group P12_1_1 with cell dimensions: a = 61.954, b = 69.984, and c = 63.858 Å with a 51.74 KDa dimer in the asymmetric unit. The structure was refined to about 2.08 Å with R_work_ = 0.24 and R_free_ = 0.26. PDB entry 8A1Q of which CTD-CCD is complexed with PIR (32) was used as the starting model for the CTD-CCD + EKC110 structure (PDB with code 8T5B).

### MD simulations

As a starting point for all MD simulations, we utilized the crystal structure of PIR + WT CCD-CTD (PDBID: 8A1Q) and modeled the disordered chain regions not resolved in the CCD domain of the structure: residues 145 to 148 of CCD subunit 1 and residues 141 to 147 of CCD subunit 2, using Modeller (48). Subsequently, an all-atom model for Apo CCD dimers was derived by removing PIR from its complex with the inhibitor (PDBID: 8A1Q). The initial structure for the WT CCD dimer in complex with EKC110 was derived from the CCD-CTD + PIR complex by alchemically transforming the bound PIR molecules into EKC110 by substituting a methyl group from the pyrrolopyridine-based aromatic scaffold of PIR to hydrogen. For all models, we added hydrogens to HIV-1 IN according to the protonation state of the amino acids at pH 7.0 as predicted by propKa (49) while maintaining the Mg^2+^ ions from the crystal structures. These models were then prepared for molecular simulation by solvating each system with TIP3P water molecules into a periodic box and ionizing with Na^+^ and Cl^-^ ions to achieve a concentration of 150 mM in VMD (50). The final simulation domains contained 142,889 and 142,883 atoms for the IN complexes with PIR and EKC, respectively, with overall system dimensions of 115 Å × 108 Å × 119 Å.

In addition, for the 1 μs MD simulations of CCD_Y99H/A128T_-CTD complexed with PIR and EKC, we used the mutator plugin in VMD to introduce the mutations in the described structure (PDBID: 8A1Q), and then, we derived coordinates for the ALLINIs into the CTD-CCD binding pocket in the same position as the WT structures and kept the Mg^2+^ ions. Structures for the CCD_Y99H/A128T_-CTD in complex with ALLINIs were then solvated and ionized following the same procedure described in the previous paragraph. The fully solvated models contained 144,082 atoms for the CCD_Y99H/A128T_-CTD in complex with PIR and EKC and system dimensions of 117 Å × 111 Å × 117 Å.

Prior to MD simulations, we performed the following equilibration procedure for all wild-type and Y99H/A128T IN complex systems (51). First, we energy minimized the solvent and ions around the protein while constraining the positions of protein and ligand atoms with a harmonic constant of 100 kcal/mol; the minimization procedure used the conjugate gradient scheme and was extended until the gradient converged to values below 10 kcal mol^−1^Å^−1^. Next, we thermalized the solvent and ions by slowly raising the temperature of the simulation domain from 50 K to 310 K at a rate of 0.5 K/ps while maintaining the constraints on the positions of protein and ligand atoms. A second energy minimization step followed, in which the restraints in the positions of protein and ligand atoms were released, allowing the positions of all atoms in the system to be optimized until the conjugate gradient converged to values below 10kcal mol^−1^ Å^−1^. This minimization procedure was followed by a second thermalization step where the positions of the protein backbone atoms were harmonically restrained with a light harmonic constant of 10 kcal/mol and the temperature of the simulation domain was slowly raised from 50 K to 310 K at a rate of 0.5 K/ps. Subsequently, we performed NPT equilibration simulations while the restraints on protein backbone atoms were slowly released at a rate of 2 kcal/mol/ns from 10 kcal/ to 0 kcal/mol over 5ns. For the equilibration simulations, we maintained the temperature at 310K using a Langevin thermostat with a thermal coupling constant of 1 ps^−1^ and a pressure of 1 atm via a Nose-Hoover barostat with a period of 100 fs and decay time of 50 fs.

After conducting the equilibration procedure, we performed 1 μs MD simulations in the NPT ensemble at a temperature of 310 K and pressure of 1 atm using the Langevin thermostat and Nose-Hoover barostat with the same parameters as above. Throughout all simulations, we used a 2 fs timestep and periodic boundary conditions. Long-range electrostatic interactions were calculated using the particle mesh Ewald method with a short-range cutoff of 12 Å and switching parameter of 10 Å. Throughout all MD and FEP simulations, the coordination number between the two Mg^2+^ ions and protein within 5 Å in the CCD was constrained using the *coordNum* function in the Colvar module (52) of NAMD. All simulations were performed using the CHARMM36m force field parameters for proteins (53), and the TIP3P model for water molecules (54). Force field parameters for both PIR and EKC were derived by analogy from the CHARMM general force field version 4.5 using CGenFF2.5 (55, 56). In total, summing the simulations for the wild-type and Y99H/A128T IN dimer systems in complex with PIR and EKC110 or in the absence of ALLINIs, we compile a cumulative sampling of 5μs. All canonical MD simulations were performed in the NAMD3 molecular dynamics simulation engine taking advantage of GPU-accelerated computing (57).

### Binding pocket volume and orientation measurements

From the trajectories of 1 μs MD simulations for WT CCD-CTD and CCD_Y99H/A128T_-CTD in complexes with PIR and EKC110, we calculated the internal volume of the CCD-CTD binding pocket by defining it as an outer shell of the protein atoms within 10 Å of the ALLINI bound and using *measure volinterior* plugin in VMD (58) for fuzzy-boundary volume detection with a grid spacing of 1 Å, isovalue of 0.8, resolution of 5.5, and 64 rays cast by every voxel. Volumes reported are calculated using the 90-th percentile confidence threshold.

To quantify the displacement of the CTD domain from the CCD_Y99H/A128T_-PIR-CTD and CCD_Y99H/A128T_-EKC110-CTD complexes simulations with respect to their WT CCD-ALLINI-CTD counterparts, we measured the distance between the centers of mass of the CTD domains in the mutant and WT complexes after 1 μs molecular sampling by using the measure center command in VMD (50) and using the molecular mass of the atoms as weight. In addition, we used the package orient to calculate the principal axis of inertia of the CTD domains in the WT and mutant complexes and computed the angles of rotation between the axes in both complexes. We denote θ, Φ, and Ψ, as the angles of rotation between the first, second, and third principal axes of the CTD domains of the wild-type and mutant complexes throughout the text.

### FEP calculations

Alchemical FEP calculations were applied to the Y99H/A128T IN resistance mutations to quantify their effect on the binding of PIR and EKC110. Starting from the CCD_Y99H/A128T_-ALLINI-CTD and WT CCD + ALLINI complex crystal structures obtained in the present work, a dual-topology structure including the WT and mutant residues was created using the mutator plugin in VMD (50). These structures were then prepared for molecular simulation by solvating them in a TIP3P water box and ionizing them with Na^+^ and Cl^-^ ions to a salt concentration of 150 mM. Furthermore, all systems were subjected to the same equilibration procedure as the 1 μs MD simulations, described above, followed by a 15 ns post-restraint release equilibration step in the NVT ensemble at a temperature of 310K maintained via a Langevin thermostat with a coupling constant of 1 ps^−1^. All FEP calculations were performed in the NVT ensemble with a temperature of 310 K and a Langevin thermostat coupling constant of 1 ps^−1^. All other simulation specifications and force field parameters were kept the same as in the long-scale MD simulations. All FEP calculations were performed using the NAMD2.14 molecular dynamics simulation engine (59).

The relative free energy differences were calculated using a thermodynamic cycle (Fig. S8B), where the vertical arms yield the free energy difference corresponding to the binding of PIR or EKC110 to WT or mutant IN ($\Delta G_{wt}^{bind}$ and $\Delta G_{mut}^{bind}$), whereas the horizontal arms yield the free energy difference due to the residue substitution in the unbound HIV-1 IN and ALLINI-bound HIV-1 IN states ($\Delta G^{free}$ and $\Delta G^{comp}$). In this manner, the relative free energy can be computed as


$$\Delta \Delta G=\Delta G^{comp}-\Delta G^{free}=\Delta G_{wt}^{bind}-\Delta G_{mut}^{bind}$$

Here, we determined the free energy differences corresponding to the horizontal arms of the thermodynamic cycle ($\Delta G^{free}$ and $\Delta G^{comp}$) via alchemical transformation of the residues using a dual-topology paradigm (60) in molecular dynamics simulations. In the dual-topology paradigm, we use a hybrid energy function:


$$H\left(x,p;\lambda \right)=H_{0}\left(x,p\right)+\lambda H_{wt.resid}\left(x,p\right)+\left(1-\lambda \right)H_{mut.resid}\left(x,p\right)$$

in which λ is a coupling parameter connecting the physical wild-type (λ = 1) and mutant (λ = 0) states through alchemical states (0 < λ < 1). The FEP simulations were performed in a bidirectional approach by running 20 sequential equally spaced λ-windows in the forward direction from the WT to mutant IN followed by a simulation in the backward direction, from the mutant to WT IN (Fig. S8A) employing a soft-core van der Waals radius-shifting coefficient of 4.0 Å. Each window of the alchemical transformation encompassed 1 ns of simulation, of which 0.2 ns were used to equilibrate the simulation domain while the following 0.8 ns of sampling were used for the free energy calculations.

The free energy differences due to the residue substitution in the ALLINI-unbound IN system ($\Delta G^{free}$) and in the ALLINI-bound IN system ($\Delta G^{comp}$) were calculated from the forward and backward trajectories using the Bennet acceptance ratio estimator (61) as implemented in the ParseFEP plugin (62) in VMD (50). All FEP simulations were repeated in three independent replicates, the relative free energy differences (ΔΔ*G*) reported are the result of averaging the calculated ΔΔ*G* for the three independent replicates and the error bars represent the standard deviation between independent measurements (Fig. S8C).

## Data Availability

The data presented in this article are available from the corresponding authors upon reasonable request. The refined models and the associated X-ray diffraction data have been deposited into the Protein Data Bank under accession codes 8S9Q (PIR + CCD), 8T5A (PIR + CCD_Y99H/A128T_), 8D3S (EKC110 + CCD), 8T52 (EKC110 + CCD_Y99H/A128T_), and 8T5B (CTD-CCD + EKC110).

## SUPPLEMENTAL MATERIAL

Supplemental material mbio.00465-24-s0001.docx Supplemental tables, figures, and text.

ASM does not own the copyrights to Supplemental Material that may be linked to, or accessed through, an article. The authors have granted ASM a non-exclusive, world-wide license to publish the Supplemental Material files. Please contact the corresponding author directly for reuse.
