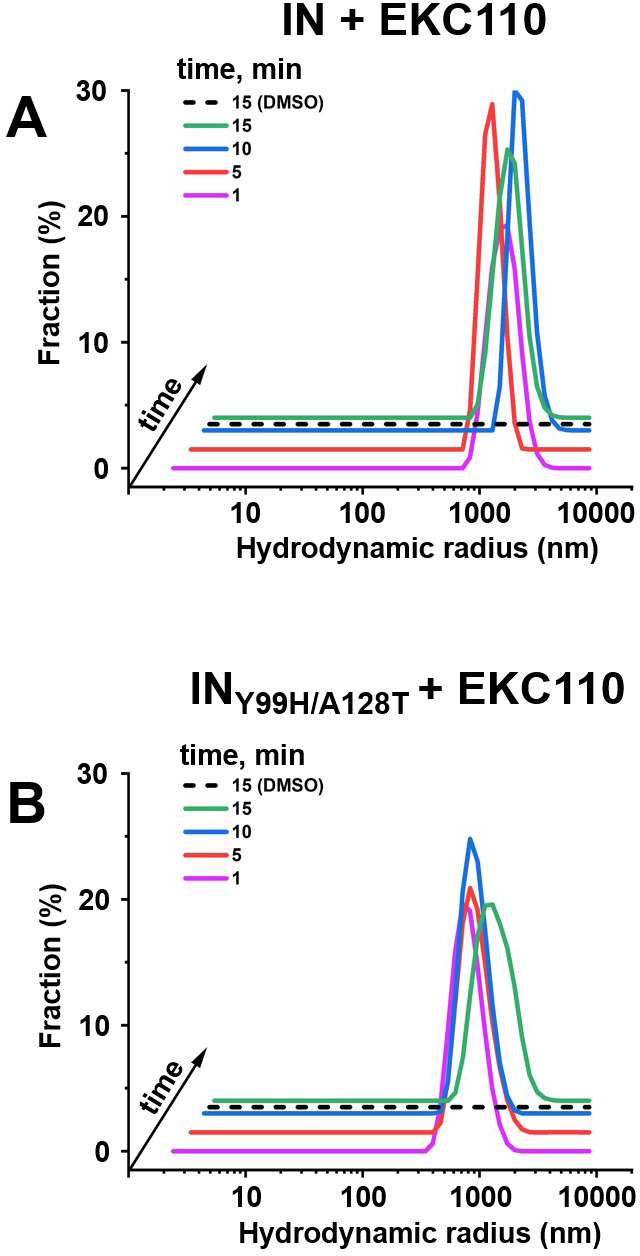# Erratum for Dinh et al., “The structural and mechanistic bases for the viral resistance to allosteric HIV-1 integrase inhibitor pirmitegravir”

**DOI:** 10.1128/mbio.03106-25

**Published:** 2025-11-04

**Authors:** Tung Dinh, Zahira Tber, Juan S. Rey, Seema Mengshetti, Arun S. Annamalai, Reed Haney, Lorenzo Briganti, Franck Amblard, James R. Fuchs, Peter Cherepanov, Kyungjin Kim, Raymond F. Schinazi, Juan R. Perilla, Baek Kim, Mamuka Kvaratskhelia

## ERRATUM

Volume 15, no. 11, e00465-24, 2024, https://doi.org/10.1128/mbio.00465-24. Page 14: Figure 9 should appear as shown in this erratum. In the published article, Fig. 9 was a duplicate of Fig. 2. We apologize for this error, which did not change the final result.

**Fig 9:**