# Supplementary material for: The structural and mechanistic bases for the viral resistance to allosteric HIV-1 integrase inhibitor pirmitegravir
Source: mBio. 2024 Oct 15;15(11):e00465-24. doi: 10.1128/mbio.00465-24 (PMC11559089; doi:10.1128/mbio.00465-24)
Supplement: Supplemental material — Supplemental tables, figures, and text. [file mbio.00465-24-s0001.docx]

**Supplementary Materials**

**The structural and mechanistic bases for the viral resistance to allosteric HIV-1 integrase inhibitor pirmitegravir**

**The structural and mechanistic bases for the viral resistance to allosteric HIV-1 integrase inhibitor pirmitegravir**

**Authors:** Tung Dinh^1^*, Zahira Tber^#2^, Juan S. Rey^3^, Seema Mengshetti^2^, Arun S. Annamalai^1^, Reed Haney^1^, Lorenzo Briganti^1^, Franck Amblard^2^, James R. Fuchs^4^, Peter Cherepanov^5^, Kyungjin Kim^6^, Raymond F. Schinazi^2^, Juan R. Perilla^3^, Baek Kim^2^*, Mamuka Kvaratskhelia^1^*

^1^ Division of Infectious Diseases, University of Colorado Anschutz Medical Campus, Aurora, Colorado, USA.

^2^ Center for ViroScience and Cure, Laboratory of Biochemical Pharmacology, Department of Pediatrics, Emory University School of Medicine, and Children’s Healthcare of Atlanta, Atlanta, Georgia, USA.

^3^ Department of Chemistry and Biochemistry, University of Delaware, Newark, Delaware, USA.

^4^ College of Pharmacy, The Ohio State University, Columbus, Ohio, United States.

^5^ Chromatin Structure & Mobile DNA Laboratory, The Francis Crick Institute, London, United Kingdom.

^6^ ST Pharm Co., Ltd., Seoul, South Korea.

^#^ Contributed equally.

*Corresponding authors: [mamuka.kvaratskhelia@cuanschutz.edu](mailto:mamuka.kvaratskhelia@cuanschutz.edu); [tung.dinh@cuanschutz.edu](mailto:tung.dinh@cuanschutz.edu); [baek.kim@emory.edu](mailto:baek.kim@emory.edu).

**Table S1. Data collection for the crystal structures.**

| **complexes** | **WT CCD + PIR** | **CCD_Y99H/A128T_ + PIR** | **WT CCD + EKC110** | **CCD_Y99H/A128T_ + EKC110** | **CTD-CCD + EKC110** |
| --- | --- | --- | --- | --- | --- |
| **PDB ID** | **8S9Q** | **8T5A** | **8D3S** | **8T52** | **8T5B** |
| **X-Ray source** | **Rigaku Micromax 007** | **Rigaku Micromax 007** | **Rigaku Micromax 007** | **Rigaku Micromax 007** | **ALS 4.2.2** |
| **Software** | **XDS** | **XDS** | **XDS** | **XDS** | **XDS** |
| **Wavelength** | **1.54178** | **1.54178** | **1.54178** | **1.54178** | **1.00003** |
| **Space group** | **P 3_1_ 2 1** | **P 3_1_ 2 1** | **P 3_1_ 2 1** | **P 3_1_ 2 1** | **P 1 2_1_ 1** |
| **Unit cell dimension**  **a, b, c (Å)**  **a, b, g (Å)** | **72.0, 72.0, 65.9**  **90, 90, 120** | **71.8, 71.8, 66.0**  **90, 90, 120** | **72.5, 72.5, 66.2**  **90, 90, 120** | **72.2, 72.2, 66.4**  **90, 90, 120** | **62.0, 70.0, 63.9**  **90, 100.732, 90** |
| **Resolution (Å)** | **31.17 - 2.26 (2.341 - 2.26)** | **31.56 - 1.933 (2.003 - 1.933)** | **29.28 - 1.855 (1.921 - 1.855)** | **31.28 - 2.075 (2.149 - 2.075)** | **46.72 - 2.08 (2.154 - 2.08)** |
| **No. total reflection** | **224392 (13449)** | **21086 (78)** | **29323 (456)** | **21042 (356)** | **59212 (4594)** |
| **No. unique reflection** | **9576 (941)** | **10545 (39)** | **14677 (240)** | **11041 (284)** | **31574 (2807)** |
| **R*_merge_*** | **0.288 (1.947)** | **0.08091 (0.3051)** | **0.02835 (0.2928)** | **0.02927 (0.5911)** | **0.06936 (0.9956)** |
| **R*_pim_*** | **0.05948 (0.5318)** | **0.08091 (0.3051)** | **0.02835 (0.2928)** | **0.02927 (0.5911)** | **0.06936 (0.9956)** |
| **CC1/2** | **0.997 (0.506)** | **0.993 (0.788)** | **0.999 (0.913)** | **0.999 (0.365)** | **0.997 (0.32)** |
| **I/sI** | **8.67 (0.82)** | **7.97 (1.64)** | **18.04 (1.87)** | **28.93 (0.98)** | **6.22 (0.52)** |
| **Completeness** | **99.94 (100.00)** | **69.77 (2.63)** | **83.52 (13.78)** | **87.16 (22.95)** | **97.17 (84.72)** |
| **Multiplicity** | **23.4 (14.3)** | **2.0 (2.0)** | **2.0 (1.9)** | **1.9 (1.3)** | **1.9 (1.6)** |
| Data were collected from a single crystal. Value in parentheses are from the highest-resolution shell. | | | | | |

**Table S2: Refinement statistics of the crystal structures.**

| **complexes** | **WT CCD + PIR** | **CCD_Y99H/A128T_ + PIR** | **WT CCD + EKC110** | **CCD_Y99H/A128T_ + EKC110** | **CTD-CCD + EKC110** |
| --- | --- | --- | --- | --- | --- |
| **PDB ID** | **8S9Q** | **8T5A** | **8D3S** | **8T52** | **8T5B** |
| **No. reflection used in refinement** | **9575 (941)** | **10544 (39)** | **14556 (235)** | **10880 (280)** | **31455 (2733)** |
| **No. reflection used for R_free_** | **946 (95)** | **568 (2)** | **1449 (27)** | **1104 (28)** | **1998 (165)** |
| **R_work_ (%)** | **0.2551** | **0.2567 (0.4078)** | **0.2820** | **0.2607** | **0.2403 (0.3660)** |
| **R_free_ (%)** | **0.2930** | **0.2784 (0.8347)** | **0.3028** | **0.3091** | **0.2643 (0.3986)** |
| **No. non-hydrogen atoms**  **Protein**  **Ligand/ion**  **Water** | **1130**  **1028**  **65**  **67** | **1102**  **1028**  **65**  **39** | **1081**  **1040**  **62**  **7** | **1108**  **1028**  **62**  **46** | **3399**  **3247**  **126**  **82** |
| **Wilson B-factor** | **36.03** | **25.20** | **29.75** | **27.21** | **35.79** |
| **Average B-factor**  **Protein**  **Ligand/ion**  **Water** | **43.19**  **42.99**  **38.17**  **48.80** | **31.44**  **31.44**  **30.30**  **35.17** | **36.95**  **37.12**  **33.26**  **28.78** | **36.69**  **36.73**  **32.57**  **38.76** | **43.64**  **43.89**  **34.46**  **41.65** |
| **R.m.s deviations**  **Bond length (Å)**  **Bond angle (^o^)** | **0.002**  **0.35** | **0.011**  **0.87** | **0.011**  **1.22** | **0.001**  **0.35** | **0.211**  **3.40** |
| **Ramachandran**  **Favored (%)**  **Allowed (%)**  **Outliers (%)** | **98.41**  **1.59**  **0.0** | **98.41**  **1.59**  **0.0** | **98.41**  **1.59**  **0.00** | **98.41**  **1.59**  **0.0** | **95.99**  **3.26**  **0.75** |
| **Rotamer outliers (%)** | **0.00** | **0.00** | **1.80** | **0.00** | **1.78** |
| **Clash score** | **1.43** | **3.81** | **0.94** | **0.48** | **4.23** |

**Fig S1.** (A) The schematic representation of IN domains. (B) The V-shaped cavity at the CCD-CCD dimer interface in the absence (left) and presence of PIR (right). (C) ALLINI induced head to tail interactions between CCD-CCD of one dimer and the CTD of another dimer, which lead to the formation of non-functional protein polymers (1).

**FIG S2.** Virion productions for WT and indicated mutant viruses. Representative immunoblots showing capsid (p24), matrix (MA) and IN levels of WT and the mutant viruses. HIV-1 virions were produced in HEK293T cells by transfecting 2 µg full-length pNL4-3 containing WT or dug-resistant mutant INs. 48 h post-transfection viruses present in supernatant were collected, clarified, and filtered through 0.45 µm filter. Viral supernatants were analyzed by western blotting using anti-HIV1 p55 + p24 + p17 antibody (abcam catalog no. ab63917) and monoclonal anti-HIV1 integrase antibody [IN-2] (abcam catalog no. ab66645) in ChemiDoc XRS + System (bio-rad).

**FIG S3.** Antiviral activities of PIR against WT and IN mutant viruses. HIV-1 virions were prepared in the presence of indicated concentrations of PIR or DMSO as control in HEK293T cells. Target cells (HeLa TZM-bl) were infected with pretreated virions in the presence of PIR at same concentrations or DMSO as control. 48 h post infection infectivity was measured by luciferase assay. Effective concentration (EC_50_) values of PIR were calculated using the Origin software (OriginLab, Inc.) and are reported in Table 1.


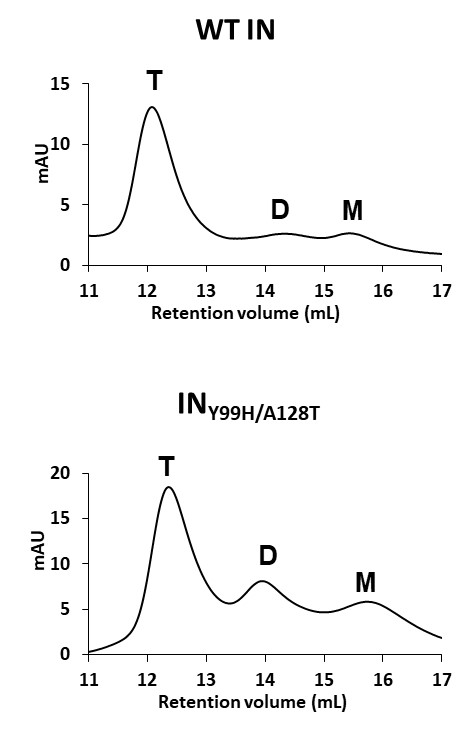


**FIG S4.** Analytical SEC of full-length WT IN (A) and IN_Y99H/A128T_ (B). Elution chromatograms (A_280_) of indicated recombinant HIV-1 IN from a Superdex-200 10/30 column are shown. The expected elution volumes for the tetrameric (T), dimeric (D), and monomeric (M) INs are indicated above each chromatogram.

**FIG S5.** The crystal structure of EKC110 (blue) bound to the V-shaped pocket at the CCD dimer interface (green). Shown are bidentate hydrogen bonding established by the EKC110 pharmacophore carboxylate with backbone amides of Glu170 (2.7 Å) and His171 (2.9 Å). Furthermore, the Thr174 side chain hydrogen bonds with the oxygen of the *tert*-butoxy moiety (3.3 Å) and the carboxylate (2.8 Å) of EKC110.

**
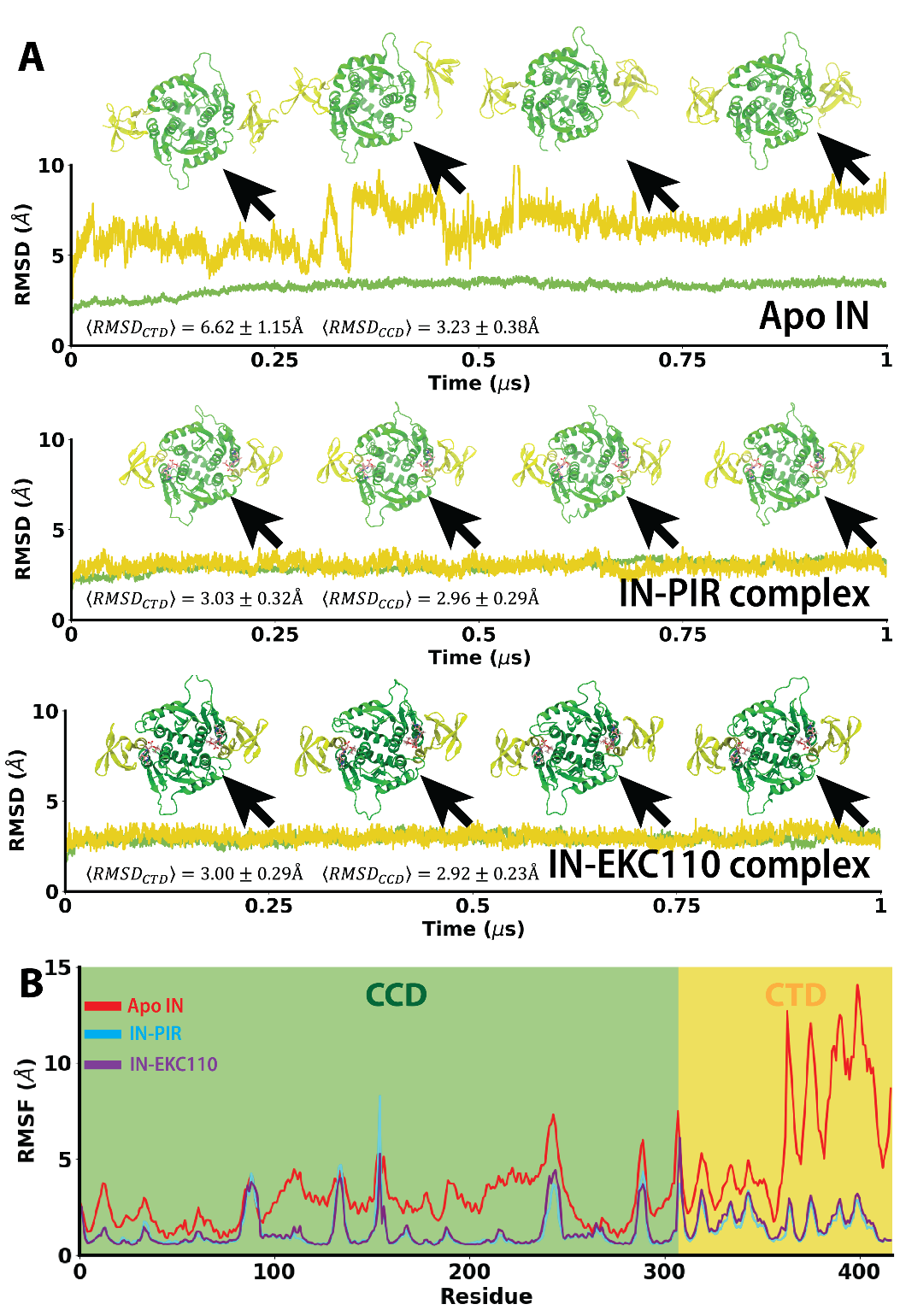
**

**FIG S6.** ALLINI effects on the CTD-CCD interactions. (A) Root means squared deviations (RMSD) calculated for the CCD (green) and CTD (yellow) domains of IN in Apo, PIR bound and EKC110 bound complexes though 1 μs MD simulation. Snapshots of IN CTD-CCD+ALLINI conformations are displayed every 250 ns of the MD simulation as indicated by the black arrows. B) Root mean squared fluctuation (RMSF) calculated for each residue in the absence of ALLINIs (red) or in the complex with PIR (cyan) or EKC110 (purple).

**
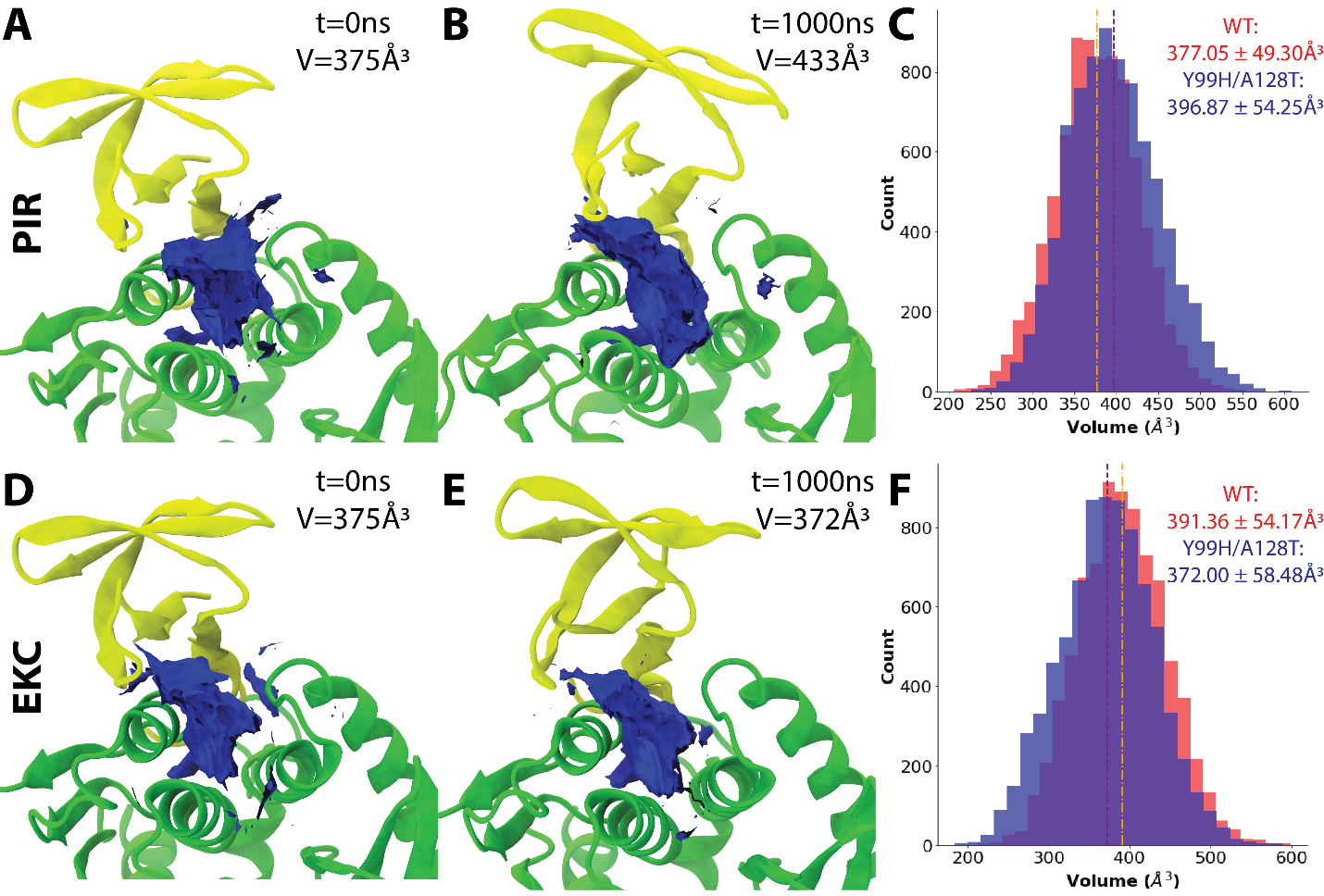
**

**FIG S7.** The CCD-ALLINI-CTD interface volume analyses. (A, D) Initial CCD-CTD interface volume for CCD_Y99H/A128T_ in the complex with PIR (A) or EKC110 (D). (B, E) CCD-CTD interface internal volume after 1 μs simulation for CCD_Y99H/A128T_ in the complex with PIR (B) or EKC (E). IN CCD and CTD domains are colored in green and yellow, respectively, and the CCD-CTD interface volume is in blue. (C, F) Internal volume distributions of the CCD-CTD interface for WT (blue) and the Y99H/A128T mutant (red) INs in complex with PIR (c) or EKC (f) over 1 μs of simulation.

**
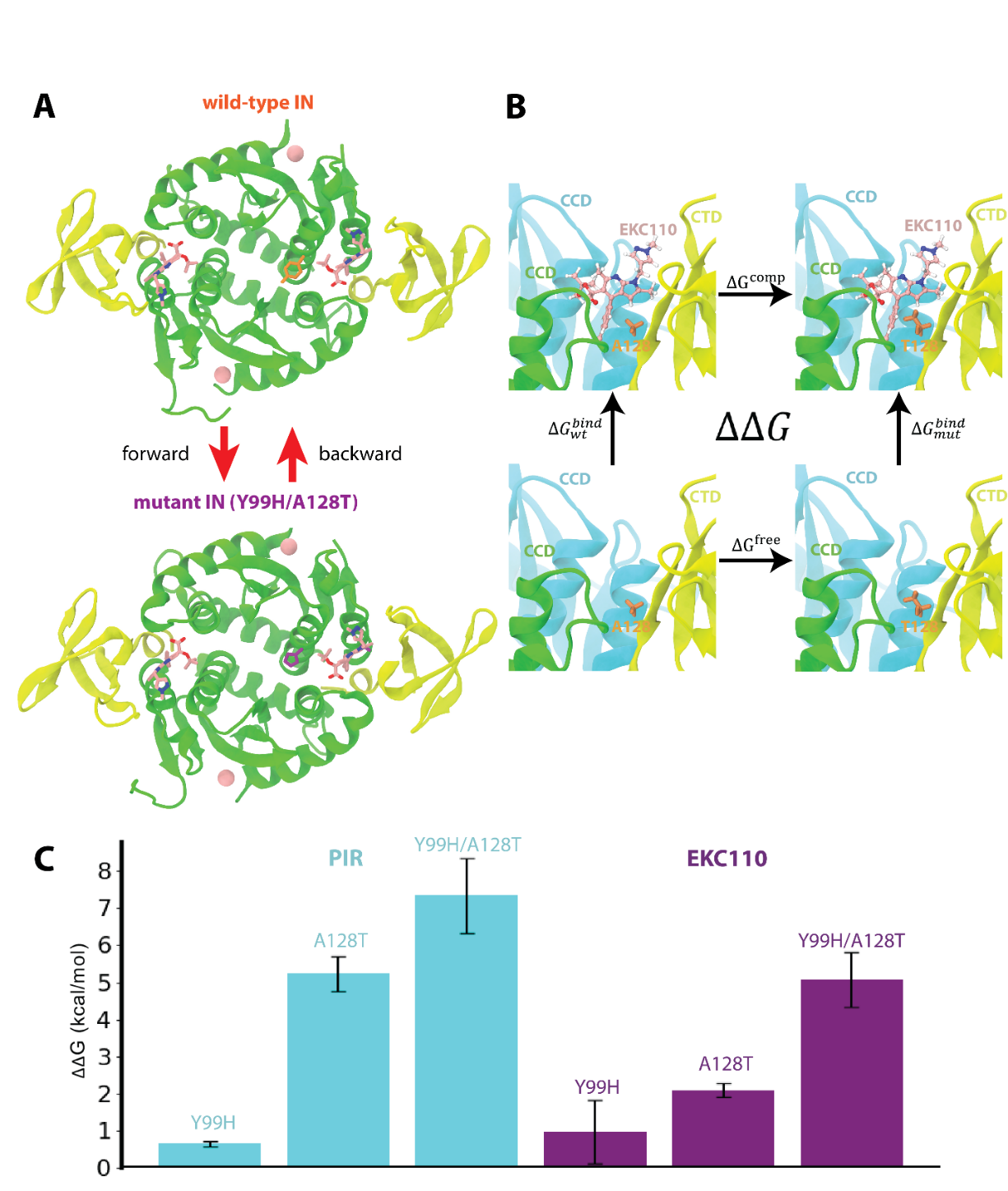
**

**FIG S8.** The free energy perturbation calculations. (A) The alchemical substitutions of WT IN residues to the drug-resistant mutants were made in a forward (WT to mutant) and backward (mutant to WT) path. Snapshots for the initial and final states of the FEP trajectories for the Y99H/A128T IN mutations on the CTD-CCD + EKC110 are shown. CTD and CCD are colored yellow and green, respectively, while the Mg^2+^ is colored pink. WT Tyr99 and Ala128 residues are colored orange, while the mutated His99 and Thr128 are colored purple. (B) Thermodynamic cycle used to calculate relative free energy differences caused by CTD-CCD IN residue substitutions in the complex with EKC110. The horizontal paths represent the alchemical residue substitutions, and the vertical paths represent the ALLINI binding. IN CTD is colored in yellow and CCD subunits 1 and 2 are colored in green and cyan, respectively. (C) Relative free energy differences induced by the Y99H, A128T and Y99H/A128T mutations in the presence of PIR and EKC110 at the CTD-CCD interface.

**FIG S9.** SPR analysis of EKC110 binding to WT CCD and CCD_Y99H/A128T_. Representative sensorgrams for EKC110 binding to WT CCD (A) vs CCD_Y99H/A128T_ (B). EKC110 concentrations are indicated. The dissociation constant (*K_D_*) and the Hill coefficient (n) for EKC110 + CCD (C) and EKC110 + CCD_Y99H/A128T_ (D) were determined using the Hill equation.

**FIG S10.** Affinity pull-down assays to probe EKC110 induced CCD-CTD interactions. Lane 1: molecular weight markers; Lanes 2 - 4: loads of His_6_-CCD (lane 2), His_6_-CCD_Y99H/A128T_ (lane 3), and tag-less CTD (lane 4); Lanes 5 - 7: affinity pull-down using Ni beads of CTD alone (lane 5, control), His_6_-CCD + CTD (lane 6), His_6_-CCD_Y99H/A128T_ + CTD (lane 7) in the absence of EKC110; Lanes 8 - 10: affinity pull-down using Ni beads of CTD + EKC110 (lane 8, control), His_6_-CCD + EKC110 + CTD (lane 9), His_6_-CCD_Y99H/A128T_ + EKC110 + CTD (lane 10).

**Scheme 1.** Synthesis of compound **19** (EKC110)

**Synthesis of compound 19 (EKC110)**

**General Procedures.** Anhydrous solvents were purchased from Aldrich Chemical Co., Inc. (Milwaukee, WI). All commercially available reagents were used without further purifications. Reagents were purchased from commercial sources. All the reactions were carried out under nitrogen in oven-dried glassware unless otherwise noted. Thin layer chromatography was performed on Analtech GHLF silica gel plates. Column chromatography was accomplished on Combiflash Rf200 or via reverse-phase high performance liquid chromatography. ^1^H, ^13^C, ^19^F, and ^31^P NMR spectra were recorded on a Bruker Ascend 400 spectrometer at 25 °C (400 MHz, 101 MHz, 377 MHz and 162 MHz) as noted and residual proton solvent signals were used as internal standards. Deuterium exchange and decoupling experiments were utilized to confirm proton assignments. NMR processing was performed with MestReNova version 10.0.2-15465. Signal multiplicities are represented by s (singlet), d (doublet), dd (doublet of doublets), t (triplet), q (quadruplet), br (broad), bs (broad singlet), m (multiplet). Coupling constants (*J*) are in hertz (Hz). Mass spectra were determined on a Micromass Platform LC spectrometer using electrospray ionization. Purity of final compounds was determined to be >95%, using UPLC analyses performed on a Waters Acquity UPLC System with a Kinetex LC column (2.1 mm Å, 50 mm, 1.7 μm, C18, 100 Å) and further supported by clean NMR spectra. Mobile phase flow was 0.4 mL/min with a 1.20 min gradient from 95% aqueous media (0.05% formic acid) to 95% CH_3_CN (0.05% formic acid) and a 4.5 min total acquisition time. Photodiode array detection was from 190 to 360 nm.

**Diethyl 2-(1-aminoethylidene) malonate (2)**

To a solution of diethyl malonate **1** (19.05 mL, 124 mmol) and acetonitrile (6.46 mL, 124 mmol) in 1,2-dichloroethane (DCE) (80 mL) under nitrogen atmosphere was slowly added tin(IV) chloride (SnCl_4_) (31.9 mL, 272 mmol) over 30 min and then the mixture was heated to 125 °C for 2.5 h. The mixture was concentrated to a paste and then dissolved in acetone (350 mL), transferred to a beaker, vigorously stirred and saturated Na_2_CO_3_/water (250 mL) was added dropwise to pH 9-10. The slush was filtered through a bed of celite, stirring the surface of the Celite® to facilitate filtering. The filter cake was washed with dichloromethane (4×200 mL). The aqueous phase was separated from the filtrate and the organic phase was dried (Na_2_SO_4_), concentrated and purified by column chromatography (90/10 to 80/20 hexane/ethyl acetate) to provide the desired compound **2** (20 g, 80%);  ^1^H NMR (400 MHz, CDCl_3_): *δ* 8.88 (s, 1H), 5.20 (s, 1H), 4.16 (dq, *J* = 19.8, 7.1 Hz, 4H), 2.12 (s, 3H), 1.26 (dt, *J* = 12.6, 7.1 Hz, 6H); ^13^C NMR (101 MHz, CDCl_3_): *δ* 169.0, 168.6, 163.4, 93.2, 60.4, 59.7, 21.9, 14.4, 14.3; MS (EI, m/e) = 202 (M+1).

**4-(Bromomethyl)-1-methyl-1*H*-pyrazole (4)**

To a stirred solution of (1-methyl-1*H*-pyrazole-4-yl)methanol **3** (1.5 g, 13.37 mmol) in glacial acetic acid (7.5 ml) was added 33 % HBr in acetic acid (18 mL, 66.96 mmol) and the mixture was refluxed for 5 h. The solvent was removed under reduced pressure. The residue was crystallized from DCM and Et_2_O to afford the desired compound **4** (1.5 g, 64%); ^1^H NMR (400 MHz, D_2_O) *δ* 8.00 (s, 2H), 4.50 (s, 2H), 4.00 (s, 3H); ^13^C NMR (101 MHz, D_2_O) *δ* 135.1, 132.6, 122.8, 53.5, 37.9; MS (EI, m/e) = 198 (M+Na^+^).

**5-Methyl-1-((1-methyl-1*H*-pyrazol-4-yl)methyl)pyrrolidin-2-one (6)**

To a solution of 5-methyl-2-pyrrolidinone **5** (755 mg, 7.61 mmol) in THF (30 mL) was added NaH (60 %, 610 mg, 15.22 mmol) at 0 ^o^C. The mixture was stirred at rt for 30 min, then a solution of 4-(bromomethyl)-1-methyl-1*H*-pyrazole (**4**) (2 g, 11.41 mmol) in DMF (1 mL) was added. The resulting mixture was stirred at rt overnight. The reaction was quenched with NH_4_Cl (20 ml), diluted with ethyl acetate (20 mL) and then concentrated under vacuum. The residue was dissolved in ethyl acetate (50 mL) and washed with H_2_O (10 mL). The organic layer was dried over MgSO_4_ and evaporated under reduced pressure. The crude residue was dissolved in acetonitrile (10 mL) and washed with hexane (10 mL x 2). The acetonitrile layer was concentrated and dried over MgSO_4_ to afford the desired compound (**6**) (1 g, 68%); ^1^H NMR (400 MHz, MeOD) *δ* 7.56 (s, 1H), 7.40 (s, 1H), 4.63 (d, *J =* 15.2 Hz, 1H), 4.03 (d, *J =* 15.2 Hz, 1H), 3.84 (s, 3H), 3.71 – 3.55 (m, 1H), 2.48 – 2.12 (m, 3H), 1.76 – 1.55 (m, 1H), 1.23 (d, *J =* 6.3 Hz, 3H); ^13^C NMR (101 MHz, MeOD) *δ* 177.3, 139.8, 131.5, 118.2, 54.8, 38.8, 35.1, 31.2, 27.6, 19.9; MS (EI, m/e) = 194 (M+1).

**Diethyl (Z)-2-(1-((5-methyl-1-((1-methyl-1H-pyrazol-4-yl)methyl)pyrrolidin-2-ylidene)amino) ethylidene)malonate (7)**

To a solution of **6** (3.2g, 16.55 mmol) in 1,2-dichloroethane (DCE) (20 mL) under nitrogen atmosphere was added POCl_3_ (2.32 mL, 24.82 mmol) dropwise over 15 minutes. The resulting mixture was stirred at ambient temperature for 1 h before addition of diethyl 2-(1-aminoethylidene)malonate (**2**) (4.33 g, 21.52 mmol). After heating the mixture at 40 °C for 22 h, saturated NaHCO_3_/water was carefully added (20 mL) and the mixture was stirred at rt for 1 h then extracted with dichloromethane (3 x 40 mL). The combined organic phase was washed with brine, dried (Na_2_SO_4_), and concentrated under vacuum. The product was purified by flash chromatography using hexane/ethyl acetate (80/20 to 50/50) to afford the desired compound **7** (2.80 g, 45 %); ^1^H NMR (400 MHz, CDCl_3_) *δ* 7.39 (s, 1H), 7.35 (s, 1H), 4.61 (d, *J* = 15.1 Hz, 1H), 4.15 (q, *J* = 7.0 Hz, 2H), 4.11 – 3.99 (m, 2H), 3.93 (d, *J* = 15.2 Hz, 1H), 3.83 (s, 3H), 3.50 (q, *J* = 7.0 Hz, 1H), 2.51 – 2.30 (m, 2H), 2.21 (s, 3H), 2.09 – 1.99 (m, 1H), 1.53 – 1.42 (m, 1H), 1.23 (t, *J* = 7.0 Hz, 3H), 1.16 (ddd, *J* = 15.1, 7.0, 1.2 Hz, 6H); ^13^C NMR (101 MHz, CDCl_3_) *δ* 167.4, 166.6, 166.1, 158.9, 139.0, 130.2, 116.9, 110.6, 60.4, 60.3, 54.5, 53.5, 38.9, 35.5, 28.3, 27.1, 22.2, 19.6, 14.3; HRMS-ESI (m/z) [M+H]^+^ calcd. for C_19_H_29_N_4_O_4_: 377.2111 found: 377.2182

**Ethyl 4-hydroxy-2,6-dimethyl-1-((1-methyl-1*H*-pyrazol-4-yl)methyl)-2,3-dihydro-1** **-pyrrolo[2,3-*b*]pyridine-5-carboxylate (8)**

To a solution of **7** (1.3 g, 3.45 mmol) in DMF (9 mL) was added sodium ethoxide (21% wt./EtOH, 3.88 mL, 10.37 mmol) and the mixture was placed in a pre-heated oil bath at 100 °C and stirred for 4 h. The reaction was cooled to 0 ^o^C and the pH was adjusted by addition of 1 N HCl to pH = 8-9. The solvents were removed under vacuum; the residue was dissolved in dichloromethane (3 x 20 mL) and washed with water. The organic layers were combined and dried over MgSO_4_. The solvent was removed under vacuum. The residue was purified by flash chromatography using hexane/ethyl acetate (50/50 to 40/60) to afford the desired compound **8** (797 mg, 70%); ^1^H NMR (400 MHz, CDCl_3_) *δ* 11.86 (s, 1H), 7.36 (s, 1H), 7.25 (s, 1H), 4.91 (d, *J* = 15.3 Hz, 1H), 4.35 (q, *J =* 7.0 Hz, 2H), 4.04 (d, *J* = 15.4 Hz, 1H), 3.82 (s, 3H), 3.80 – 3.74 (m, 1H), 3.08 – 3.01 (m, 1H), 2.65 (s, 3H), 2.43 (ddd, *J* = 15.7, 7.0, 0.9 Hz, 1H), 1.39 (t, *J* = 7.0 Hz, 3H), 1.27 (d, *J* = 7.0 Hz, 3H); ^13^C NMR (101 MHz, CDCl_3_) *δ* 171.8, 164.4, 163.4, 162.3, 139.3, 129.5, 117.4, 101.7, 101.45, 61.2, 55.3, 39.0, 35.8, 31.1, 27.8, 19.8, 14.4; HRMS-ESI (m/z) [M+H]^+^ calcd. for C_17_H_23_N_4_O_3_: 331.1692, found: 331.1764.

**Ethyl 4-hydroxy-2,6-dimethyl-1-((1-methyl-1*H*-pyrazol-4-yl)methyl)-1*H*-pyrrolo[2,3-*b*]pyridine-5-carboxylate (9)**

A solution of **8** (1 g, 3.02 mmol) in toluene (15 mL) and 2,3-dichloro-5,6-dicyano-1,4-benzoquinone (DDQ) (1.03 g, 4.5 mmol) was stirred at rt for 20 min. The solvent was removed under reduced pressure. The residue was purified by flash chromatography using hexane/ethyl acetate (50/50 to 40/60) to afford the desired compound **9** (575 mg , 58%); ^1^H NMR (400 MHz, CDCl_3_-*d*) *δ* 12.61 (s, 1H), 7.39 (s, 1H), 7.16 (s, 1H), 6.31 (s, 1H), 5.26 (s, 2H), 4.46 (q, *J* = 7.1 Hz, 2H), 3.80 (s, 3H), 2.83 (s, 3H), 2.38 (s, 3H), 1.46 (t, *J* = 7.1 Hz, 3H); ^13^C NMR (101 MHz, CDCl_3_) δ 172.8, 163.1, 155.2, 138.3, 134.5, 129.1, 118.8, 107.34, 101.8, 97.1, 61.6, 39.1, 36.0, 28.1, 14.4, 13.1; HRMS-ESI (m/z) [M+H]^+^ calcd. for C_17_H_21_N_4_O_3_: 329.1535, found: 329.1605.

**Ethyl 2,6-dimethyl-1-((1-methyl-1*H*-pyrazol-4-yl)methyl)-4-(((trifluoromethyl)sulfonyl) oxy)-1H-pyrrolo[2,3-*b*]pyridine-5-carboxylate (10)**

To a solution of **9** (356 mg, 1.08 mmol) in dichloromethane (4 mL) was added triethylamine (0.218 ml, 1.62 mmol) at 0 ^o^C followed by dropwise addition of Tf_2_O (0.194 mL, 1.18 mmol). The mixture was stirred at rt for 1h before being concentrated under vacuum. The crude product was purified by flash chromatography using hexane/ethyl acetate (50/50) to afford the desired compound **10** (497 g, 90 %); ^1^H NMR (400 MHz, CDCl_3_) *δ* 7.39 (s, 1H), 7.19 (s, 1H), 6.28 (s, 1H), 5.29 (s, 2H), 4.43 (q, *J* = 7.1 Hz, 2H), 3.80 (s, 3H), 2.75 (s, 3H), 2.45 (s, 3H), 1.41 (t, *J* = 7.1 Hz, 3H); ^13^C NMR (101 MHz, CDCl_3_) *δ* 165.8, 151.5, 150.3, 145.2, 139.6, 138.3, 129.2, 118.6 (q, *J* = 320.5 Hz), 117.9, 114.5, 111.0, 96.1, 62.2, 39.1, 36.4, 24.1, 14.1, 13.3; ^19^F NMR (377 MHz, CDCl_3_) *δ* -73.45; HRMS-ESI (m/z) [M+H]^+^ calcd. for C_18_H_20_F_3_N_4_O_5_S: 461.1028, found: 461.1151.

**Ethyl 4-(4-chlorophenyl)-2,6-dimethyl-1-((1-methyl-1*H*-pyrazol-4-yl)methyl)-1*H*-pyrrolo[2,3-*b*]pyridine-5-carboxylate (11)**

To a solution of **10** (500 mg, 1.08 mmol) in dry DMF (5 mL) were added 4-chlorophenylboronic acid (194 mg, 1.24 mmol) and potassium carbonate (447 mg, 3.24 mmol), The reaction mixture was degassed with a stream of nitrogen for 5 min and then tetrakis-triphenylphosphine palladium (Pd(PPh_3_)_4_) (125 mg, 0.108 mmol) was added thereto. The reaction was degassed one more time with a stream of nitrogen, and heated under nitrogen atmosphere for 4 h at 100°C. The reaction solution was cooled to room temperature. The insoluble substances were removed by filtration through a celite pad. The filtrate was concentrated under reduced pressure, and then the residue was purified using silica gel column chromatography hexane-ethyl acetate (60/40 to 50/50) to give target compound **11** (364 mg, 80%); ^1^H NMR (400 MHz, CDCl_3_) *δ* 7.47 – 7.33 (m, 5H), 7.20 (s, 1H), 6.02 (s, 1H), 5.32 (s, 2H), 4.10 (q, *J* = 7.1 Hz, 2H), 3.79 (s, 3H), 2.72 (s, 3H), 2.39 (s, 3H), 1.02 (t, *J* = 7.1 Hz, 3H); ^13^C NMR (101 MHz, CDCl_3_) *δ* 170.1, 148.4, 147.7, 138.3, 138.2, 137.8, 136.5, 134.0, 130.0, 129.1, 128.6, 121.0, 118.6, 117.1, 97.9, 61.1, 39.0, 36.0, 23.3, 13.8, 13.1; HRMS-ESI (m/z) [M+H]^+^ calcd. for C_23_H_24_ClN_4_O_2_: 423.1510, found: 423.1582.

**(4-(4-Chlorophenyl)-2,6-dimethyl-1-((1-methyl-1*H*-pyrazol-4-yl)methyl)-1*H*-pyrrolo[2,3-*b*]pyridin-5-yl)methanol (12)**

To a solution of compound **11** (370 mg, 0.87 mmol) in anhydrous dichloromethane (10 mL) under nitrogen atmosphere was added a 1 M DIBAL/hexane solution (5 mL, 5 mmol) over 5 min at -78°C. The resulting solution was stirred for 2 h at the same temperature before slow addition of a saturated aqueous solution of NH_4_Cl (10 mL). The mixture was then diluted with dichloromethane (4 mL) and stirred for 30 min while slowly raising the temperature to rt. The mixture was filtered over celite. The cake was washed twice with dichloromethane (10 mL). The organic layer was separated, and the aqueous layer was extracted with dichloromethane (10 mL x2). The organic layers were combined, washed with brine (10 mL) and dried with anhydrous MgSO_4_, and the solvent was concentrated under reduced pressure. The residue was purified using silica gel column chromatography hexane/ethyl acetate (60/40 to 50/50) to give compound **12** (270 mg, 82%). ^1^H NMR (400 MHz, MeOD) *δ* 7.45 – 7.39 (m, 4H), 7.38 (d, *J* = 0.7 Hz, 1H), 7.20 (s, 1H), 5.87 (s, 1H), 5.30 (s, 2H), 4.62 (s, 2H), 3.76 (s, 3H), 2.79 (s, 3H), 2.36 (s, 3H); ^13^C NMR (101 MHz, MeOD) *δ* 151.5, 146.9, 140.6, 138.2, 136.5, 136.3, 133.7, 130.9, 129.2, 128.4, 122.9, 118.9, 118.2, 97.9, 59.6, 39.0, 35.8, 22.8, 22.7, 13.1, HRMS-ESI (m/z) [M+H]^+^ calcd. for C_21_H_22_ClN_4_O: 381.1404, found: 381.1472.

**4-(4-Chlorophenyl)-2,6-dimethyl-1-((1-methyl-1*H*-pyrazol-4-yl)methyl)-1*H*-pyrrolo[2,3-*b*]pyridine-5-carbaldehyde (13)**

To a solution of compound **12** (0.31 g, 0.82 mmol) in anhydrous dichloromethane (5 mL) was added pyridinium chlorochromate (PCC) (0.35 g, 1.63 mmol) and silica gel (0.245 g, 70% by weight of PCC) at 0 ^o^C, under nitrogen atmosphere. The resulting mixture was stirred at rt for 3 h and the reaction was filtered. The residue was washed with dichloromethane (10 mL) and the filtrate was concentrated under reduced pressure. The residue was purified using silica gel column chromatography dichloromethane /methanol (99/1 to 95/5) to give compound **13** (0.175 g, 56%); ^1^H NMR (400 MHz, CDCl_3_) δ 10.06 (s, 1H), 7.47 – 7.43 (m, 3H), 7.38 – 7.32 (m, 2H), 7.29 (s, 1H), 6.02 (s, 1H), 5.35 (s, 2H), 3.81 (s, 3H), 2.95 (s, 3H), 2.41 (m, 3H). ^13^C NMR (101 MHz, CDCl_3_) *δ* 192.8, 153.9, 148.0, 145.7, 138.4, 138.3, 134.6, 134.1, 131.3, 129.2, 128.6, 121.0, 118.2, 118.1, 98.6, 39.0, 36.0, 25.5, 13.1; HRMS-ESI (m/z) [M+H]^+^ calcd. for C_21_H_20_ClN_4_O: 379.1247, found: 379.1319.

**2-(4-(4-Chlorophenyl)-2,6-dimethyl-1-((1-methyl-1*H*-pyrazol-4-yl)methyl)-1*H*-pyrrolo[2,3-*b*]pyridin-5-yl)-2-((trimethylsilyl)oxy)acetonitrile (14)**

To a solution of compound **13** (0.175 g, 0.46 mmol) in dichloromethane (5 mL) was added ZnI_2_ (0.19 g, 0.60 mmol) at 0 ^o^C under nitrogen atmosphere followed by addition of trimethylsilyl cyanide (0.23 mL, 1.85 mmol) over 5 min. The resulting mixture was stirred for 1 h at 0 ^o^C and 3 h at 25 ^o^C. The reaction was diluted with dichloromethane (10 mL), washed with water (10 mL), dried over anhydrous MgSO_4_, and the solvent was concentrated under reduced pressure to give the target compound **14** (0.221 g, 100% crude yield) which was used in the next reaction without further purification.

**Methyl 2-(4-(4-chlorophenyl)-2,6-dimethyl-1-((1-methyl-1*H*-pyrazol-4-yl)methyl)-1*H*-pyrrolo[2,3-*b*]pyridin-5-yl)-2-hydroxyacetate (15)**

To a solution of compound **14** (0.2 g, 0.40 mmol) in methanol (10 mL) was added sulfuric acid (0.89 mL, 16.7 mmol) dropwise at 0 °C. The reaction mixture was then stirred at 75 °C for 24 h before being cooled to 0 °C and neutralized with 2N NaOH to pH 7.5. Solvents were removed under reduced pressure. The mixture was diluted with ethyl acetate (10 mL) and washed with water (10 mL). The aqueous phase was extracted with ethyl acetate (5 mL x 2) and the combined organic layers were dried over Na_2_SO_4_ and concentrated under vacuum. The resulting residue was purified by silica gel column chromatography using hexane/ethyl acetate (90/10 to 30/70) to provide **15** (0.050 g, 27%) along with starting material **14** (0.070 g); ^1^H NMR (400 MHz, MeOD) δ 7.56 – 7.37 (m, 5H), 7.32 (s, 1H), 5.81 (s, *J* = 1.1 Hz, 1H), 5.37 (s, 1H), 5.33 (s, 2H), 3.76 (s, 3H), 3.62 (s, 3H), 2.64 (s, 3H), 2.35 (s, 3H); ^13^C NMR (101 MHz, MeOD) δ 175.7, 151.7, 147.8, 142.5, 139.0, 138.8, 137.4, 135.1, 132.6, 132.0, 130.8, 129.5, 123.8, 120.3, 119.8, 99.0, 70.2, 52.9, 38.8, 36.6, 22.9, 13.0; HRMS-ESI (m/z) [M+H]^+^ calcd. for C_23_H_24_ClN_4_O_3_: 439.1459, found: 439.1470.

**Methyl 2-(4-(4-chlorophenyl)-2,6-dimethyl-1-((1-methyl-1*H*-pyrazol-4-yl)methyl)-1*H*-pyrrolo[2,3-*b*]pyridin-5-yl)-2-oxoacetate (16)**

To a solution of compound **15** (0.050 g, 0.114 mmol) in dichloromethane (DCM) (5 mL) at 0 °C was added in two portions Dess-Martin periodinane (DMP) (0.063 g, 0.148 mmol). The resulting mixture was stirred at rt for 2 h before being concentrated under vacuum and purified by chromatography using hexane/ethyl acetate (90/10 to 40/60) to provide **16** (0.043 g, 87% yield); ^1^H NMR (400 MHz, CDCl_3_) δ 7.47 – 7.41 (m, 3H), 7.37 – 7.31 (m, 2H), 7.27 (s, 1H), 6.08 (d, *J* = 1.1 Hz, 1H), 5.35 (s, 2H), 3.83 (s, 3H), 3.39 (s, 3H), 2.74 (s, 3H), 2.42 (d, *J* = 1.1 Hz, 3H); ^13^C NMR (101 MHz, CDCl_3_) δ 190.2, 163.4, 151.5, 148.1, 140.7, 138.7, 138.4, 135.1, 135.0, 131.5, 129.4, 128.9, 122.4, 118.3, 117.3, 98.1, 52.7, 39.1, 36.1, 24.0, 13.3; HRMS-ESI (m/z) [M+H]^+^ calcd. for C_23_H_22_ClN_4_O_3_: 437.1302, found: 437.1415.

**Methyl (*S*)-2-(4-(4-chlorophenyl)-2,6-dimethyl-1-((1-methyl-1*H*-pyrazol-4-yl)methyl)-1*H*-pyrrolo[2,3-*b*]pyridin-5-yl)-2-hydroxyacetate (17**)

To a solution of compound **16** (0.043 g, 0.098 mmol) in toluene (4 mL) was added (*R*)-1-methyl-3,3-diphenylhexahydropyrrolo[1,2-*c*][1,3,2]oxazaborole ((*R*)-Me-CBS, 0.024 mL, 0.024 mmol, 1 M in toluene) under nitrogen atmosphere. The mixture was cooled to -35 °C (EtOH/dry ice) and then catecholborane (0.30 mL, 0.295 mmol, 1 M in THF) was slowly added over ∼30 min. The mixture was kept at -35 °C for 30 min and then allowed to warm to 0 °C in ∼ 2 h. 2M Na_2_CO_3_/water (4 mL) was added followed by EtOAc (10 mL) and the mixture was stirred for 5 min at rt. Water was added and the organic phase was washed with 1 M NaOH/water (4 mL), dried over Na_2_SO_4_, and concentrated. The residue was purified using silica gel column chromatography hexane/ethyl acetate (90/10 to 30/70) to provide compound **17** (0.020 g, 46%). ^1^H NMR (400 MHz, MeOD) *δ* 7.58 – 7.38 (m, 5H), 7.33 (s, 1H), 5.83 (d, *J* = 1.1 Hz, 1H), 5.36 (s, 3H), 3.79 (s, 3H), 3.65 (s, 3H), 2.64 (s, 3H), 2.39 (d, *J* = 1.0 Hz, 3H); ^13^C NMR (101 MHz, MeOD) *δ* 175.7, 151.7, 147.9, 142.5, 139.0, 138.8, 137.4, 135.2, 132.6, 132.0, 130.8, 129.5, 123.8, 120.4, 119.9, 99.0, 70.2, 52.9, 38.8, 36.6, 22.9, 13.0; HRMS-ESI (m/z) [M+H]^+^ calcd. for C_23_H_24_ClN_4_O_3_: 439.1459, found 439.1535.

**Methyl (*S*)-2-(*tert*-butoxy)-2-(4-(4-chlorophenyl)-2,6-dimethyl-1-((1-methyl-1*H*-pyrazol-4-yl)methyl)-1*H*-pyrrolo[2,3-*b*]pyridin-5-yl)acetate (18)**

To a solution of **17** (0.020 g, 0.045 mmol) in tert-butyl acetate (0.489 mL, 3.65 mmol) was added dropwise perchloric acid (15 μL, 0.182 mmol) and the mixture was stirred at rt for 3 h. Saturated NaHCO_3_/water (3 mL) was added slowly and then extracted with ethyl acetate (10 mL). The organic phase was washed with brine, dried, concentrated and purified by chromatography using hexane/ethyl acetate (90/10 to 40/60) to provide compound **18** (0.016 g, 72%); ^1^H NMR (400 MHz, MeOD) *δ* 7.58 – 7.51 (m, 3H), 7.46 – 7.41 (m, 2H), 7.35 (s, 1H), 5.84 (d, *J* = 1.2 Hz, 1H), 5.36 (d, *J* = 2.2 Hz, 2H), 5.34 (s, 1H), 3.79 (s, 3H), 3.76 (s, 3H), 2.65 (s, 3H), 2.39 (d, *J* = 1.0 Hz, 3H), 0.92 (s, 9H); ^13^C NMR (101 MHz, MeOD) *δ* 175.7, 152.1, 147.7, 141.1, 139.1, 138.7, 137.6, 135.3, 132.8, 132.7, 130.9, 129.6, 129.5, 124.6, 120.4, 119.6, 98.9, 76.8, 71.2, 52.9, 38.8, 36.6, 28.3, 23.6, 13.0 ; HRMS-ESI (m/z) [M+H]^+^ calcd. for C_27_H_31_ClN_4_O_3_: 495.2085, found: 495.2159.

**(*S*)-2-(*tert*-Butoxy)-2-(4-(4-chlorophenyl)-2,6-dimethyl-1-((1-methyl-1*H*-pyrazol-4-yl)methyl)-1*H*-pyrrolo[2,3-*b*]pyridin-5-yl)acetic acid (19)**

To a solution of compound **18** (0.016, 0.032 mmol) in THF (3 mL) was added 4N NaOH (24 μL) and methanol (24 μL). The mixture was stirred for 18 h at 25 ^o^C and 4N HCl (24 μL) was added to neutralize the mixture. The resulting mixture was concentrated under vacuum and the residue was purified by column chromatography using dichloromethane/methanol (99/ 1 to 85/15) to give compound **19** (0.010 g, 66%); ^1^H NMR (400 MHz, MeOD) *δ* 7.70 (m, 1H), 7.57 – 7.50 (m, 2H), 7.48 – 7.43 (m, 1H), 7.42 (s, 1H), 7.34 (d, *J* = 0.8 Hz, 1H), 5.86 (d, *J* = 1.1 Hz, 1H), 5.36 (d, *J* = 4.8 Hz, 2H), 5.31 (s, 1H), 3.78 (s, 3H), 2.71 (s, 3H), 2.38 (d, *J* = 1.0 Hz, 3H), 0.91 (s, 9H); ^13^C NMR (101 MHz, MeOD) *δ* 152.2, 147.7, 141.1, 139.1, 138.5, 137.7, 135.3, 133.0, 132.7, 130.9, 129.6, 129.4, 125.2, 120.4, 119.5, 99.0, 76.8, 71.0, 38.8, 36.6, 28.3, 23.7, 13.0 ; HRMS-ESI (m/z) [M+H]^+^ calcd. for C_27_H_31_ClN_4_O_3_: 481.1928, found: 481.1999.

**References:**

1. Gupta K, Turkki V, Sherrill-Mix S, Hwang Y, Eilers G, Taylor L, McDanal C, Wang P, Temelkoff D, Nolte RT, Velthuisen E, Jeffrey J, Van Duyne GD, Bushman FD. 2016. Structural Basis for Inhibitor-Induced Aggregation of HIV Integrase. PLoS Biol 14:e1002584.
